# Young relicts and old relicts: a novel palaeoendemic vertebrate from the Australian Central Uplands

**DOI:** 10.1098/rsos.160018

**Published:** 2016-10-05

**Authors:** Paul M. Oliver, Peter J. McDonald

**Affiliations:** 1 Division of Evolution, Ecology and Genetics, Research School of Biology and Centre for Biodiversity Analysis, The Australian National University, Building 116, Daley Road, Acton, Australian Capital Territory 2601, Australia; 2 Flora and Fauna Division, Department of Land Resource Management, Alice Springs, Northern Territory 0870, Australia

**Keywords:** aridification, climate change, evolutionary refugia, gecko, relict, Pliocene

## Abstract

Climatic change, and in particular aridification, has played a dominant role in shaping Southern Hemisphere biotas since the mid-Neogene. In Australia, ancient and geologically stable ranges within the vast arid zone have functioned as refugia for populations of mesic taxa extirpated from surrounding areas, yet the extent to which relicts may be linked to major aridification events before or after the Pliocene has not been examined in detail. Here we use molecular phylogenetic and morphological data to show that isolated populations of saxicoline geckos in the genus *Oedura* from the Australian Central Uplands, formerly confounded as a single taxon, actually comprise two divergent species with contrasting histories of isolation. The recently resurrected *Oedura cincta* has close relatives occurring elsewhere in the Australian arid biomes with estimated divergence dates concentrated in the early Pliocene. A new taxon (described herein) diverged from all extant *Oedura* much earlier, well before the end of the Miocene. A review of data for Central Uplands endemic vertebrates shows that for most (including *Oedura cincta*), gene flow with other parts of Australia probably occurred until at least the very late Miocene or Pliocene. There are, however, a small number of palaeoendemic taxa—often ecologically specialized forms—that show evidence of having persisted since earlier intensification of aridity in the late Miocene.

## 1. Introduction

Since the Miocene, arid climates have expanded and intensified across the Southern Hemisphere [1–3]. In response, pre-existing mesic lineages have adapted, persisted in evolutionary refugia or become extinct [4–6]. Aridification has been particularly intense and widespread in Australia, and the extant biota of the vast and continuous Australian arid zone (AAZ) displays a strong signature of climate-driven diversification and biological turnover since the Miocene [2,5,7,8]. Even lineages which have radiated within the arid zone and are now widespread, are hypothesized to have contracted to climatic refugia during hyper-arid glacial maxima [5]. However, there are a number of geologically distinctive and stable habitats within the AAZ that are believed to have functioned as evolutionary refugia for mesic-adapted taxa through the expansion of severe aridity [9]. Especially prominent are blocks of geologically stable and ancient upland country and rocky ranges with associated clusters of endemic lineages or isolated populations, including (from west to east) the Pilbara, Central Ranges, MacDonnell Ranges, Flinders Ranges and Selwyn Ranges [5,10–12].

Phylogenetic investigation of putative relict taxa endemic to ranges in arid Australia provides opportunities to understand how and when major environmental changes shaped biotic turnover across central Australia, and also assess what factors may have predisposed lineages to long persistence [11,13–15]. Recent palaeo-climatic analyses have provided compelling evidence that the overall trajectory towards increasing aridity across the Southern Hemisphere (and especially Australia) since the Late Miocene was punctuated by a sustained warm mesic interval during the early Pliocene [16]. This suggests two broad timeframes of origin for relictual taxa: (i) isolation by Miocene aridification and predating the Pliocene mesic period, or (ii) wide distributions during the warm-wet Pliocene, with subsequent contraction and isolation during the cool-dry Pleistocene. The first hypothesis predicts divergences from sister lineages that date back well into the Miocene (more than 5 Ma), while the latter predicts more recent divergences (less than 5 Ma).

The MacDonnell Ranges Bioregion (MRB) lies right at heart of the vast AAZ, is particularly isolated from the ameliorating climatic influences of the oceans, and has some of the highest levels of localized plant and animal endemism in the AAZ [17–19]. Two additional upland rocky regions nearby, the Central Ranges Bioregion (CRB) to the south, and the poorly known Murchison Davenport Ranges Bioregion (MDRB) to the northeast also have isolated and putatively relictual taxa. We hereafter refer to these systems collectively as the ‘Central Uplands’. While it was long assumed that many Central Uplands endemics are ancient mesic relicts from an initial onset of aridity during the Miocene [9,20], recent research, especially on plants, has supported more recent Plio-Pleistocene range contraction or even anthropogenic dispersal [15,21].

Here, we build on recent analyses of a lineage of Australian geckos with Gondwanan ancestry (the velvet geckos: *Oedura*) [12,14,22] in order to assess the diversity and origins of isolated populations in the Australian Central Uplands. Currently only one taxon (*Oedura cincta*), relatively closely related (estimated Pliocene divergences) to populations of *Oedura* occurring elsewhere in the eastern AAZ, is recognized [22]. However, a morphologically distinctive population of *Oedura* from the southern portion of the MRB has been noted since the 1970s [23]. Recent targeted fieldwork has provided the first genetic samples of this population. Using these samples, we here test: (i) whether these populations are conspecific with *Oedura cincta* elsewhere in the Central Uplands, and (ii) assess whether the divergence of different populations of *Oedura* in the Central Uplands is likely to post- or pre-date the Pliocene mesic interval. To place patterns of divergence within *Oedura* in a broader context, and to facilitate further work on Central Uplands endemism, we also present a summary of available information on the distribution, ecology, divergence timeframes and phylogenetic relationships of all known endemic or isolated vertebrate taxa and populations.

## 2. Material and methods

### 2.1. Molecular analyses

New tissue samples of *Oedura* from the Australian arid biome were accessed from recently collected material stored in Australian National University (CCM), The Museum and Art Gallery of the Northern Territory (NTM) and the South Australian Museum (SAMA). Regions of one mitochondrial gene (*ND2*–all specimens) and two nuclear genes (Phosducin and *RAG-1*, selected specimens) were amplified following protocols presented in by Oliver *et al*. [14] (see further information in the electronic supplementary material, Methods). Our final dataset included sequences from 30 new samples of *Oedura* from arid central and eastern Australia, which were aligned with publicly available data from other *Oedura* and related gecko genera ([14]; electronic supplementary material, table S1). The final dataset comprised up to 912 bp of *RAG-1*, 393 bp Phosducin and 846 bp of the mitochondrial *ND2* gene.

Phylogenetic relationships were estimated using standard maximum-likelihood (RAxML v. 8.2.4: [24]) and Bayesian (BEAST v. 1.8.0: [25]) analyses implemented on the CIPRES Science Gateway v. 3.1 for online phylogenetic analysis (https://www.phylo.org). Partitioning strategies followed recent studies of *Oedura* [12], splitting mitochondrial data by codon, while the nuclear data were partitioned into first and second codon, and third codons. Maximum-likelihood analyses were run using the default settings for RAxML on the CIPRES portal using the GTR CAT model of sequence evolution [26]. For Bayesian analyses, we used the GTR + G model for mitochondrial data, and the HKY + G model for nuclear partitions. Final MCMC analyses were run for 20 million generations (with a burn-in of 20%), and parameter estimation and branch lengths were unlinked across partitions.

Dating analyses (BEAST v. 1.8.0; [25]) focused on a reduced dataset comprised of both nuclear and mitochondrial sequence data for single exemplars of species, candidate species and operational taxonomic units (OTUs) of *Oedura*, in addition to outgroups spanning the gecko subfamily Diplodactylidae (electronic supplementary material, table S1). As outlined elsewhere, fossil or biogeographic calibrations that provide meaningful age constraints for the pygopodoid geckos are unavailable [27]. Thus, we applied two broad normally distributed secondary age priors for basal divergence events in the Diplodactylidae that are consistent with three independent fossil-calibrated studies [14,28,29], the Core Diplodactylidae (mean 35.0 Ma (6.0 s.d.)) and the New Caledonian *Pseudothecadactylus* clade (43.0 Ma (9.0 s.d.)). For all dating analyses, we used the Yule speciation prior (appropriate and widely used for relatively divergent lineages). Owing to potential issues when combining saturated mitochondrial data with deep calibrations (even after third codons have been removed) [13,30], we undertook dating analyses on both the combined dataset with third codons removed, and the nuclear alignment alone. For each data alignment, we also ran both uncorrelated-lognormal and strict clock models and compared model likelihoods by comparing AICM values as implemented in Tracer v. 1.6 [31]. In the combined dataset, the uncorrelated-lognormal model was favoured (156.2 units lower), while in the nuclear dataset the clock model was favoured (240.6 likelihood units lower).

### 2.2. Morphology

We examined material (including type material) held in the following institutions: Australian Museum (AMS), Museum Victoria (NMV), NTM, Queensland Museum (QM), SAMA and Western Australian Museum (WAM) (electronic supplementary material, Appendix S1). Measurement protocols and acronyms (see electronic supplementary material) largely follow those outlined elsewhere [22,32].

Statistical analyses were performed in R [33]. All mensural variables were log transformed and we inspected all transformed variables for heteroscedasticity, normality and influential observations using boxplots and diagnostic plots in R. Body and head measurements were corrected for body size by regressing against principle component 1 from an initial principle components analysis (PCA) to account for possible non-allometric growth [34]. Analyses of tail morphology were restricted to specimens with original tails and tail measurements were standardized against snout-to-vent length (SVL).

We initially used PCA of the size-corrected body and head length data to examine how distinctive populations were in multivariate space. These analyses did not reduce the number of variables required to explain variation, with the first three components only explaining 38% of variation, and are therefore not considered further.

For subsequent univariate analyses, we used one-way ANOVA to test for overall differences in morphology between four genetically and geographically divergent lineages of *Oedura* in the AAZ (*Oedura fimbria*, two mtDNA lineages of *Oedura cincta* and *Oedura* sp.). For mensural variables where there was an overall difference (*p* ≤ 0.05), we ran pairwise *t*-test comparisons (Bonferroni corrected) between each population.

### 2.3. Biogeographic data

We synthesized distribution records (mainly from the Atlas of Living Australia [35]) and phylogenetic data for all apparently endemic or isolated vertebrate taxa and populations from all three Central Uplands Bioregions (MRB, CRB, MDRB). Where available, we noted the estimated timeframes of divergence between endemics and nearest extant relatives occurring elsewhere in Australia. Given the wide error bars associated with molecular dating, we chose to focus on broad eras: mid-Miocene (more than 10 Ma), late Miocene (approx. 10–5.3 Ma), Pliocene (5.3–2.5 Ma) and Pleistocene (less than 2.5 Ma). We also noted the broad ecological guild of endemic taxa or populations (aquatic (i.e. dependent on water to breed), arboreal, saxicoline (with adaptations for climbing in rocky habitats), scansorial, terrestrial or fossorial), whether endemics are allopatric from known or putative sister lineages, the ecology of nearest relatives (as above), and the biome of nearest relatives (arid, temperate and monsoonal).

## 3. Results

### 3.1. Molecular diversity of Central Uplands velvet geckos

**Figure 1.**
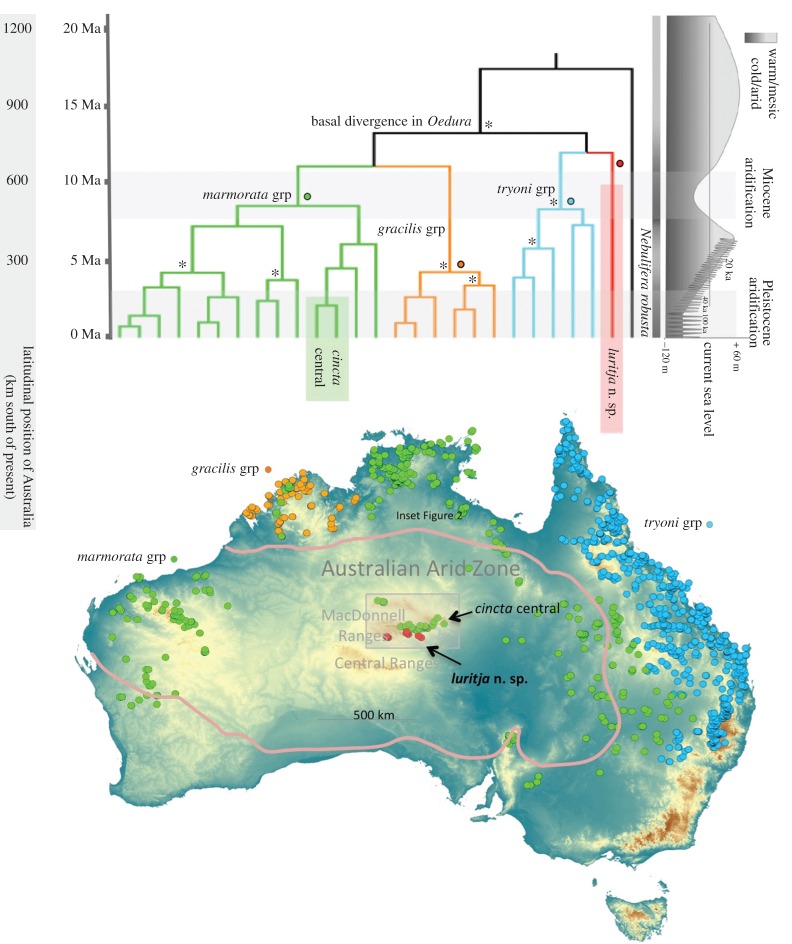
Timeframe of diversification and distribution of major clades of Australian velvet geckos (*Oedura*). Divergence time estimates derived from Bayesian analysis of two nuclear genes and secondary calibrations. Deep nodes that were well supported are highlighted with an asterisk (Bayesian posterior > 95, maximum-likelihood > 70). The two divergent lineages occurring in the Central Upland are respectively highlighted in green (*Oedura cincta*) and red (*Oedura luritja* n.sp.). Distributional data downloaded from http://ozcam.ala.org.au/. Summary diagram of Australian environmental history reproduced from [5]. Different pulses of major aridification in the late Miocene and Pliocene are highlighted.

Genetic analyses of *Oedura* identified two highly divergent and distantly related lineages within Central Uplands: (i) the recognized *Oedura cincta* (central lineage) from the granitic, quartzite, limestone and sandstone rocks of the Meerenie, Chewings and Heavitree Ranges in the north of the MRB, and (ii) a highly divergent unnamed lineage (*Oedura* n. sp.) from the Mereenie sandstones of the James Range and surrounding systems in the south of the MRB (figure 1).

Analyses based on the combined nuclear and mitochondrial alignment (third codons removed) produced older date estimates for divergence events below the calibration prior (all taxa in the main Australian radiation of diplodactylid geckos, including both lineages of Central Uplands *Oedura*) (electronic supplementary material, table S2). We interpret this as most probably an artefact of combining mitochondrial data with deep calibration nodes, and focus on date estimates from the nuclear data only. However, we also emphasize that all analyses broadly support the contention that the divergence of isolated populations of *Oedura cincta* occurred through the Plio-Pleistocene, while in contrast *Oedura* n. sp. is a much more divergent relict dating back to the early, mid- or even late Miocene.

Dating analyses, using the preferred combination of nuclear data and the correlated lognormal model, estimated very late Miocene to early Pliocene divergence of central populations of *Oedura cincta* from relatives now living in inland eastern Australia (approx. 4.3 Ma (2.1–6.8)). By contrast, *Oedura* sp. has no strongly supported sister taxon (although it did tend to associate with an assemblage of taxa from eastern Australia in both mitochondrial and nuclear phylogenies) and is estimated to have diverged from all living congeners well into the Miocene (approx. 11.6 Ma (8.1–15.9)).

**Figure 2.**
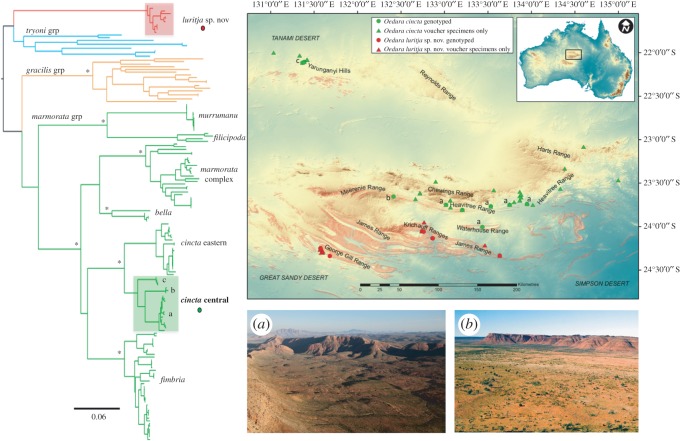
Diversity and distribution of *Oedura* in the Australian Central Uplands. Maximum-likelihood tree of all *Oedura* based on 846 bp of ND2 mitochondrial gene. Recognized taxa in the *marmorata* group are labelled, and deep intraspecific nodes that were well supported are highlighted with an asterisk (Bayesian posterior > 95, maximum-likelihood > 70). Three lineages of *Oedura cincta* (green dots labelled a, b, c) occur in a variety of rock landforms including quartzite, granites and sandstones (*a*) across the north of the MRB. *Oedura luritja* n. sp. (red dots) is restricted to a relatively continuous block of sandstones in the southern MRB (salmon shading on map, (*b*)).

Tamura–Nei distances [36] estimated using the ND2 alignment within well-sampled and widespread clades within the MRB were relatively low (*Oedura cincta* mean 0.007 (0.000–0.017); *Oedura* n. sp. 0.019 (0.000–0.035)). However, three moderately divergent mitochondrial lineages of *Oedura cincta* were identified in the greater Central Uplands region (mean inter-clade divergences 0.051–0.061); one widespread throughout the eastern and central MRB, one from a single site in the mid-north of the MRB, and an apparently isolated lineage in the ranges at the southern edge of the Tanami Desert (outside the MRB) (figure 2).

### 3.2. Morphological analyses

**Figure 3.**
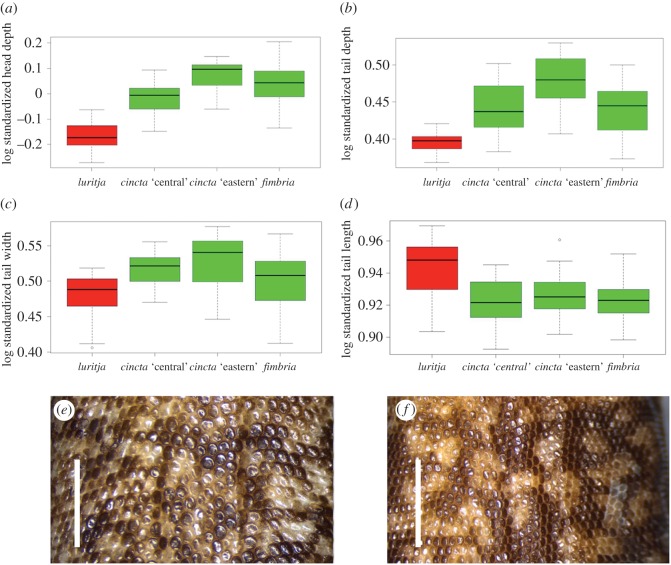
Summary morphological data. (*a–d*) Boxplots summarizing proportional differences in head depth and tails shape between major lineages of velvet gecko (*Oedura*) in the Australian arid zone, (*e*) mid-dorsal scales of adult *Oedura cincta*, (*f*) mid-dorsal scales of *Oedura luritja* n. sp. Scale bar, 5 mm.

Univariate analyses of morphology revealed consistent differences between *Oedura* n. sp. and other *Oedura* in the AAZ (figure 3*a–d*). It has a flatter head (compared with all other lineages, *p* ≤ 0.001), longer tail (compared with all other lineages, *p* ≤ 0.05), and flatter tail (compared with *O. cincta* ‘central’ and *O. fimbria*, *p* ≤ 0.01; compared with *O. cincta* ‘eastern’, *p* ≤ 0.001) (figure 3*a,b,d*). *Oedura* n. sp. also has a narrower tail than the other lineages, though this was only statistically significant when compared with *O. cincta* ‘eastern’ (*p* ≤ 0.01) (figure 3*c*). Variation in body condition (tails appear to become very thin in times of resource stress in all taxa) may explain the lack of statistical difference in tail width. No other meristic characters consistently differentiated *Oedura* n. sp. from sampled populations in the *Oedura marmorata* complex in the AAZ. A summary of all measurements for AAZ *Oedura* is provided in electronic supplementary material, table S3.

**Figure 4.**
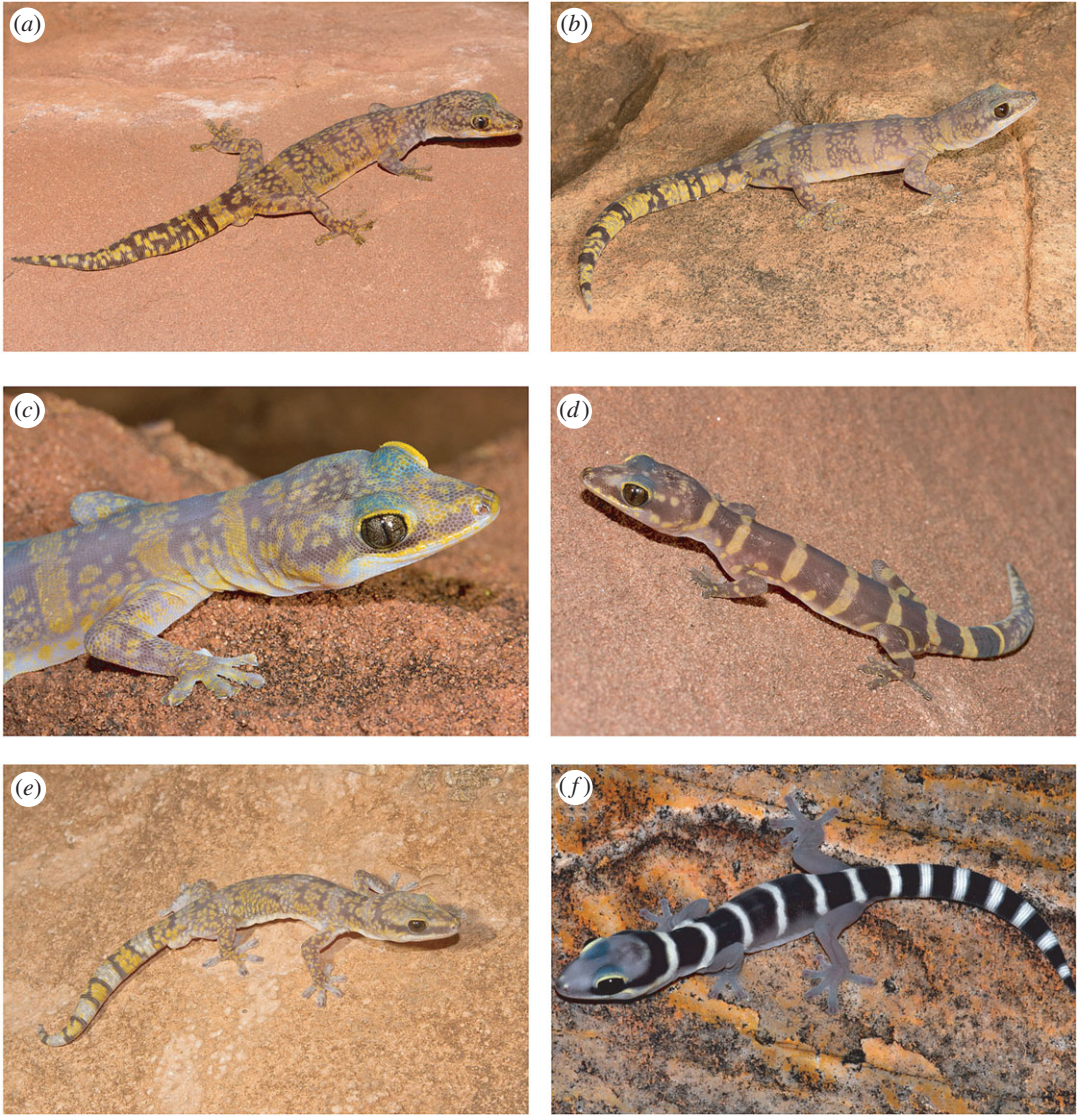
Velvet geckos (*Oedura*) from the Central Uplands in the Northern Territory, Australia. (*a*) *Oedura luritja* n. sp. holotype (CCM5974) adult male from near Boggy Hole, Finke Gorge National Park, (*b*) *Oedura luritja* n. sp. adult male from James Range, Owen Springs Reserve, (*c*) *Oedura luritja* n. sp. adult female from Palm Valley, Finke Gorge National Park, (*d*) *Oedura* n. sp. juvenile (paratype CCM5975) from near Boggy Hole, Finke Gorge NP, (*e*) *Oedura cincta* adult male from Corroboree Rock Conservation Reserve, (*f*) *Oedura cincta* juvenile from the West MacDonnell National Park. Photos (*a*–*e*) P.J.M., (*f*) Stewart MacDonald.

Morphological analyses also revealed additional diagnostic differences in the scalation and coloration of *Oedura* n. sp., in particular it is characterized by much smaller scales across the body (maximum diameter less than versus greater than 0.5 mm) (figure 3*e,f*), and the absence of strong canthal or postorbital striping (figure 4).

### 3.3. Systematics

The synonymy of the *Oedura marmorata* complex has recently been considered elsewhere [22]. Two names have been applied to populations from the Central Uplands, but are not applicable to new taxon. *Oedura greeri* Wells & Wellington [37] (holotype: AMS R87677, Mt Doreen) was described without diagnosis and is regarded as a *nomen nudum* [38]. *Oedura derelicta* Wells & Wellington [37] (holotype: NTM11413) is described based on a specimen from Jessie Gap close to Alice Springs and is currently a synonym of *Oedura cincta*.

*Oedura luritja* n. sp. (Figures 3*f* and 4*a–d*).

Mereenie velvet gecko

#### 3.3.1. Holotype

NTM R37528, field number CCM5974, adult male with regrown tail, and liver samples stored in ethanol.

#### 3.3.2. Type locality

Gorge 300 m east of north end of Boggy Hole (−24.13455, 132.86574), Finke Gorge National Park, Northern Territory, collected 5 October 2015 by P.J.M. and P.M.O.

#### 3.3.3. Paratypes

All from Northern Territory (*n* = 11). NTM R37529 (CCM5975) near Boggy Hole (−24.1351, 132.86351), Finke Gorge National Park, collected 5 October 2015; NTM R37531 (CCM5979) Palm Creek (−24.05449, 132.74246), Finke Gorge National Park; NTM R37530 (CCM5978) Palm Creek (−24.0584, 132.76151), Finke Gorge National Park, collected 6 October 2015; AMS R52143 Kings Canyon (−24.27, 131.57), Watarka National Park, collected 28 July 1975; AMS R52144–50 Reedy Springs (−24.30, 131.58), Watarka National Park, collected 28 July 1975.

#### 3.3.4. Referred material

All from Northern Territory. *Specimens* (*n* = 14): AMS R24662–71, NTM33811 Reedy Ck, George Gill Range (−24.28, 131.60); SAMA R65915, Rainbow Rocks (−24.3342, 133.6353); NTM R20681, Finke Gorge National Park (−23.95, 132.77); QMJ52883, Hermannsburg (−23.95, 132.77); AMS R52154, ‘47 mi sw of Alice Springs' (−24.27, 133.47). *Tissue samples only* (*n* = 6): CCM5973, Finke Gorge National Park (−24.13455S, 132.86574E); CCM5976, Finke Gorge National Park (−24.0584, 132.76151); CCM5977, Finke Gorge National Park (−24.05449, 132.74246); CCM6227, Kings Canyon (−24.25576, 131.57649), Watarrka National Park, collected 11 February 2015; CCM2228, Kings Canyon (−24.24966, 131.57366), Watarrka NP, collected 25 February 2015; CCM6229, Kathleen Springs (−24.33941, 131.68045), Watarrka NP, collected 26 February 2015.

#### 3.3.5. Etymology

Luritja is a collective name for people speaking several dialects of the Aboriginal Western Desert language. The western parts of the distribution of *Oedura luritja* (including Watarrka National Park) are in Luritja lands. Luritja is also believed to be derived from the Arrernte word ‘Ulerenye’ meaning foreigner or stranger, and is therefore further appropriate for such a deeply divergent lineage. Used as a noun in apposition.

#### 3.3.6. Diagnosis

A moderately large (to 99 mm SVL) species of *Oedura* with a moderately wide (HW/SVL 0.17–0.20) and flat head (HD/SVL 0.072–0.091), tail moderately long (original TL/SVL 0.65–0.87), narrow (TW/SVL 0.07–0.11), distinctly narrower than head and body and tapering gradually to a tip, rostral less than 50% divided, postcloacal spur usually single (22 out of 23 specimens), 10–16 precloacal pores in adult males, dorsal scales small (less than 0.5 mm in diameter), head brown with light flecking but with no trace of a light canthal stripe or dark-brown postorbital or nuchal stripes, and dorsal coloration of adults usually including five to six moderately well-defined light bands or transverse blotches (yellow in life) on a purplish brown background.

#### 3.3.7. Particulars of holotype

Adult male with regrown tail (figure 4*a*). Quantitative measurements in millimetres: SVL 97.0, HW 18.7, HD 8.2, HL 22.9, EN 8.3, IN 3.6, IO 7.4, EYE 5.4, TrK 43.1, ArmL 13.0, LegL 14.6, 3FW 2.8, 3TW 3.2, TL 80, TW 10, TD 6.7. Scale counts: SuL 12 (10), InF 12, CS 1/1, 3FL 8, 3TL 9, precloacal pores 11 (divided medially by 2 poreless scales).

#### 3.3.8. Description

A large (to 99 mm SVL) and moderately elongate *Oedura* (Trk/SVL 0.43–0.50); head moderately wide (HW/SVL 0.17–0.20) and flat (HD/SVL 0.072–0.091). Rostral 20–50% divided, bordered dorsally by two nasals, nasals bordered dorsally by two relatively small supranasals and 0–3 (mode 3) small intervening scales. Supralabials 10–12 to midpoint of eye, 12–15 in total; infralabials 11–15. Forelimbs and hindlimbs of moderate length (FA/SVL 0.12–0.14, TA/SVL 0.13–0.16). Subdigital lamellae moderately expanded and prominent, 7–8 under third finger, 8–9 under third toe. Distal lamellae divided, proximal lamellae undivided. Apical lamellae on terminal scansors separated from more proximal pairs. Lamellae series on fingers 2–5 and toes 2–5 flared at midpoint, at most equal in width to terminal scansors.

Original tail moderately long (TL/SVL 0.65–0.87), narrow (TW/TL 0.10– 0.15) and depressed in cross-section, tapering gradually to tip and with a slight ventral groove. Relative width and depth varies greatly depending on body condition. Caudal scalation homogeneous. Fully regrown tails similar length (TL/SVL 0.58–0.78) and width (TW/TL 0.11–0.16) to original tails.

In preservative, base coloration of dorsum dark purplish brown, generally with 5–6 distinct pale buff relatively straight and clearly defined transverse dorsal bands, or occasionally transverse series of blotches. Extensive further light buff flecking usually present between the bands, and elsewhere on the dorsal and lateral surfaces of the head, torso and limbs; light and brown pigmentation on head not forming clear lines or stripes. Two paratypes (AMS R52145 and AMS R52148) have strongly defined bands, lack extensive light flecking and have unusually large light blotches on the head and lateral edges of torso, and a single adult specimen (AMS R52143) lacks any clear dorsal bands. Venter plain light buff, with faint brownish tinge around the terminal lamellae and occasionally fine brown maculations around the infralabials. Original tail with 7–8 indistinct light bands on a brown background. Regrown tails brown with extensive and variable light flecking that does not form a distinct pattern.

In life, the basic pattern and coloration of individuals matches those of preserved specimens, however, the darker regions are purplish during the day, silvery grey at night, and light regions tend to be relatively bright yellow (figure 4*a,b*). Iris very dark brown (figure 4*c*).

Juveniles with simpler pattern of clear light transverse bands (and occasionally lateral blotches) on a plain dark-brown background, with light and dark bands becoming increasingly indistinct with size (figure 4*d*).

#### 3.3.9. Distribution and habitat

Currently known only from the sandstone ranges of the southern MRB, extending from Rainbow Valley Conservation Reserve in the east to Watarrka National Park in the west. Apparently, suitable habitat is continuous between these localities and it presumably occurs throughout the intervening region.

All specimens with associated data have been collected from sandstone and it appears to be moderately common (on suitably warm nights they can be reliably spotlighted). They are generally found in association with deep but tight cracks under exfoliating sandstone, often near exposed vertical faces, and retreat into these if threatened ([23]; P.J.M. March 2016, personal observation). Field observations indicate they are most active during the summer (daytime maxima above 35°C). In winter, they have been collected under flat exfoliating sandstone on the tops of ridges in Watarrka National Park. Gecko species observed in sympatry are *Gehyra moritzi*, *Gehyra versicolor*, *Heteronotia binoei* and *Nephrurus amyae*.

#### 3.3.10. Comparisons

Similar in overall proportions to and has been confounded with *Oedura cincta* (both central and eastern populations) but can be distinguished by its shorter rostral crease (less than 50% divided versus fully divided). Further differs from both *Oedura cincta* and *Oedura fimbria* (Western Australia) in head and tail proportions (see Results and electronic supplementary material, table S3), in its smaller body scales (mid-dorsal scales on adults < 0.5 mm wide versus > 0.5 mm wide), in generally single cloacal spur (22 out of 23 specimens examined) (versus up to 3), in generally lacking obvious light canthal stripes, brown postorbital stripes and brown nuchal bands (versus present), and in generally retaining strong and distinctly edged dorsal bands into adulthood (versus much more indistinct or absent) (figure 4).

Differs from *Oedura bella* and members of the *Oedura gemmata*-*marmorata* species complex from northern Australia in possessing a longer tail (TL/SVL 0.65–0.87 versus 0.49–0.65 and 0.53–0.63, respectively) that is also narrower (always narrower than the head versus as wide or wider), and by generally having just one clocal spur (versus 2–3).

Differs from *Oedura gracilis* (Kimberley region) by its moderately long tail (versus very long (approaching length of body)) and flared lamellae series on fingers and toes 2–5 (versus tapering); and from *Oedura filicipoda* and *Oedura murrumanu* in having narrower proximal lamellae on fingers 3–4 (not wider than the apical lamellae versus distinctly wider), and further differs from the former species in having a narrow tail that is not wider than the head and near circular in cross-section (versus wider and very flattened).

Distinguished from the remaining *Oedura* in eastern Australia (here referred to as the *tryoni* group) by its dorsal colour pattern consisting of 5–6 distinct to indistinct narrow light bands with poorly defined light flecking (versus wide pale V-shaped transverse bands in *Oedura castelnaui*, distinct dark-edged ocelli or transverse bands of varying size in *Oedura coggeri*, *Oedura monilis* and *Oedura tryoni*, or two pale bands across the nape and base of tail in *Oedura jowalbinna*). It also has a less swollen tail than *Oedura castelnaui*, and is larger (SVL up 99 mm) than *Oedura coggeri* (70 mm) and *Oedura jowalbinna* (69 mm).

### 3.4. Biogeography of Central Uplands vertebrates

The ecology and divergence dates of endemic vertebrate taxa (*n* = 19) and apparently isolated populations (*n* = 10) in the Central Uplands are summarized in table 1. Most endemic taxa (afforded unique taxonomic status) and isolated populations (currently recognized as conspecific with taxa occurring elsewhere in Australia) are clearly allopatric from extant relatives. A majority of endemics have relatives that occur in arid or seasonally arid biomes and none are closely allied to taxa in mesic forest biomes of eastern or far southwestern Australia. Published median or mean estimates of divergence age for endemic taxa are concentrated around the very late Miocene and continue through the Plio-Pleistocene. Divergence timeframes for the majority of endemic populations are not available, but given putative conspecific status may be assumed to be young. The largest number of endemic taxa are saxicoline (*n* = 6), but there are also largely terrestrial forms that are closely associated with rocky habitats (4), and a suite of aquatic (3), fossorial (4), scansorial (1) and arboreal taxa (1) that appear to be dependent on isolated microhabitats with reliable water. Endemic populations tend be less outwardly specialized (usually terrestrial) taxa with relatives that occur in habitats often widely distributed across both arid and non-arid environments.

**Table 1.** Summary of distributional, ecological and divergence data for endemic taxa and populations of vertebrates in the Australia Central Uplands. CU, Central Uplands; MRB, Macdonnell Ranges Bioregion; CRB, Central Ranges Bioregion; MD Murchison Davenport Ranges Bioregion; n.a., data not available.

species	organism	MRB	CRB	MDRB	allopatric	divergence	CU ecology	biome, relatives	ecology, relatives	references
**endemic taxa**										
*Mogurnda larapintae*	fish	x			y	n.a.	aquatic	arid	aquatic	[39]
*Pseudophyrne robinsoni*	frog		x		y	n.a.	aquatic	arid, temperate	aquatic	[40]
*Litoria gilleni*	frog	x			y	n.a.	aquatic	arid, monsoonal	aquatic	[41]
*Crenadactylus horni*	gecko	x	x	x	y	late Miocene	saxicoline	arid, pilbara	saxicoline	[13]
*Gehyra moritzi*	gecko	x		x	y	n.a.	saxicoline	ambiguous	ambiguous	[42]
*Gehyra pulingka*	gecko		x		y	n.a.	saxicoline	ambiguous	ambiguous	[42]
*Heteronotia fasciolatus*	gecko	x			y	Pliocene	saxicoline	arid, pilbara	saxicoline	[43]
*Nephrurus amyae*	gecko	x	x		y	Pleistocene	saxicoline	arid, monsoonal	saxicoline	[8]
***Oedura luritja*** n. sp	**gecko**	**x**			**y**	**mid-Miocene**	**saxicoline**	**ambiguous**	**ambiguous**	**this paper**
*Lerista frosti*	skink	x			y	Pliocene	fossorial	arid	fossorial	[44]
*Lerista speciosa*	skink		x		y	late Miocene	fossorial	arid	fossorial	[44]
*Liopholis margaretae personata*	skink	x	x		y	n.a.	saxicoline	arid, Flinders Ranges	saxicoline	[45]
*Liopholis slateri slateri*	skink	x			n.a.	n.a.	terrestrial	arid	terrestrial	n.a.
*Australotyphlops centralis*	snake	x			y	late Miocene	fossorial	arid	fossorial	[46]
*Australotyphlops fossor*	snake	x			n.a.	n.a.	fossorial	n.a.	fossorial	[47]
*Morelia spilota bredli*	snake	x			y	n.a.	arboreal	arid, temp, monsoon	arboreal	n.a.
*Ctenophorus rufescens*	dragon		x		y	n.a.	saxicoline	arid	saxicoline	[48]
*Zyzomys pedunculatus*^a^	rodent	x			y	n.a.	saxicoline	n.a.	saxicoline	n.a.
*Amytornis purnelli*	bird	x	x	x	y	Pleistocene	saxicoline	arid, Selwyn Ranges	saxicoline	[49]
**endemic populations**										
*Diplodactylus galeatus*	gecko	x			y	n.a.	saxicoline	arid	saxicoline	n.a.
*Oedura cincta*	gecko	x			y	Pliocene	saxicoline	arid	arboreal	[12]
*Strophurus intermedius*	gecko	x	x		y	n.a.	arboreal	arid	arboreal	n.a.
*Underwoodisaurus milii*	gecko	x			y	n.a.	terrestrial	arid, temperate	terrestrial	n.a.
*Ctenotus alacer*	skink	x		x	y	n.a.	terrestrial	arid	terrestrial	
*Proablepharus reginae*	skink	x	x		y	n.a.	terrestrial	arid	terrestrial	n.a.
*Tiliqua scincoides*	skink		x		y	n.a.	terrestrial	arid, temp, monsoon	terrestrial	n.a.
*Acanthophis pyrrhus*	snake	x	x	x	y	n.a.	terrestrial	arid	terrestrial	n.a.
*Pseudonaja textilis*	snake	x			y	Pleistocene	terrestrial	arid, temperate	terrestrial	[50]
*Vermicella vermiformis*	snake	x			y	n.a.	terrestrial	monsoonal	terrestrial	[51]
*Trichosaurus vulpecula vulpecula*^a^	possum	x			y	n.a.		arid, temp, monsoon	arboreal	n.a.

^a^neoendemic [52,53].

## 4. Discussion

The Central Uplands of Australia are considered a hotspot of localized endemic relicts stemming from widespread extinction or range contraction in surrounding regions [18]. In support of this contention, most endemic vertebrates in the Australian Central Uplands have allopatric sister taxa or conspecific populations occurring elsewhere in arid or semi-arid Australia (table 1). The overarching role of refugial dynamics in generating endemism is also emphasized by the lack of evidence for ecological speciation [54] in the Central Uplands (although a gecko occurring in ranges just to the north provides one possible exception [42]). A further emerging theme from recent genetic research on plants, as well as the available data we have compiled here (table 1), is that the majority endemic species and populations are not particularly old, with many showing evidence of genetic exchange with relatives occurring in more peripheral parts of Australia during the Pliocene, and in some cases even more recently [15,21].

By striking contrast, our estimates of the divergence time for *Oedura luritja* suggest much earlier diversification around the mid- to late Miocene. Even more unusually for Central Uplands endemics, this taxon also has no strongly supported sister lineage, implying both long-term persistence and widespread extinction [55]. This lineage diverged from extant relatives well before the major expansion of Australia's vast sandy deserts through the Plio-Pleistocene [2,5,56], but on a timeframe that is consistent with isolation by an earlier period of intensifying aridity in the late Miocene [16]. While the pattern shown by this taxon is currently unique, phylogenetic and distributional data suggest that a number of other relatively restricted and specialized saxicoline lizard taxa in the Central Uplands also show Miocene divergences and/or a lack of close relatives [13,42]. Thus, while a majority of Central Uplands relicts probably do postdate the Pliocene wet phase, as dated molecular phylogenies accumulate, it seems likely that a smaller number will be shown to have longer histories of isolation (especially in specialized taxa, those with low mobility and/or a high capacity to persist in localized microrefugia, e.g. [13,57]).

Based on our review of the endemic vertebrate fauna in the Central Uplands, the presence of two morphologically and ecologically similar, congeneric vertebrate relicts occurring in the same bioregion is also unusual. *Oedura luritja* has smaller scales and a generally thinner tail, characteristics respectively linked to higher rates of evaporative water loss [58,59] and a reduced capacity to store resources in arid climates [60,61]. Conversely, this species also has a relatively flat head, a trait often observed in specialized crevice dwelling lizards [62], and its known distribution is entirely restricted to the rocks of a single geological landform (Mereenie sandstones). By contrast, *Oedura cincta* has a deeper body and larger tail, a comparatively wide distribution across eastern and central Australia and includes populations that use both rocks (predominantly granite, quartzite and limestone) and trees, with genetic data suggesting gene flow across now uninhabited regions during the Plio-Pleistocene (presumably on trees along watercourses where eastern populations still exist). The regional coexistance of these two taxa could be linked to this variation in specialization and climatic tolerance, coupled with local microhabitat differences in different Central Uplands Ranges. Further physiological and ecological data are required to develop and test this hypothesis further.

In the AAZ, it is clear that upland ranges, particularly in the Pilbara region, are characterized by deeper genetic diversity and higher genetic structuring than surrounding regions [63,64]. However, most studies have focused on comparative patterns of genetic diversity in rocky regions, rather than the timing of isolation. Thus the relative contribution of periods of aridification before and after the early Pliocene to shaping patterns of relictual endemism in the Central Uplands, other arid zone refugia in Australia, and elsewhere in the Southern Hemisphere, remains to be systematically addressed. However, molecular phylogenetic analyses of other Australia arid zone taxa, including other diplodactylid gecko genera with Gondwanan affinities [13,65], subterranean diving beetles [66] and salt lake beetles [7] are increasingly suggesting that the initial isolation of at least some arid zone relicts predates the Pliocene, and was potentially concurrent with widespread aridification during the Miocene. Many taxa showing long persistence tend to be ecologically specialized (e.g. fossorial or saxicoline), or otherwise show traits linked with vulnerability to environmental change and extremes (i.e. very small size [13]), but which may also predispose them to localized persistence on areas of stable habitat within an otherwise dynamic biome.

## 5. Conclusion

Previous taxonomy of Australian velvet geckos (*Oedura*) masked the existence of two highly divergent relicts that may have been isolated by different aridification events before and after the Pliocene. Available evidence indicates that the isolation of the majority of endemic vertebrate taxa in the Central Ranges of Australia probably postdates the onset of the Pliocene, although a small number of generally specialized lizard taxa (including the new species we describe herein) show evidence of deeper Miocene divergences. Similar deeply divergent and biogeographically significant, but overlooked, relict lineages continue to be discovered in arid and seasonally arid biomes across several major landmasses [11,12,67–70]. In many of these taxa a close association with geological landforms that provide stable and protected microhabitats appears to be one key factor underpinning their persistence at very local scales, over long timescales and through major climatic changes.

## Supplementary Material

1. Supplementary methods.

## Supplementary Material

2. Supplementary Tables and Appendices: Table S1. Specimens number, locality data and genbank numbers for all samples included in genetic analyses; Table S2. Summary of results from dating analyses. Table S3; Summary of morphological data for velvet geckos in the genus Oedura from the Australian Arid Zone; Appendix S1. Summary specimens included in morphological analyses and comparisons.

## Supplementary Material

3. Treefile 1. Chronogram (nexus format) estimated using nuclear data alignment, with two partitions, Yule prior and uncorrelated lognormal model implemented in BEAST v. 1.8. 4.

## Supplementary Material

Treefile 2. Maximum-Likelihood Phylogeny (nexus format) estimated from complete mitochondrial dataset.

## Data accessibility

The supplementary tables and figures supporting this article have been uploaded as part of the electronic supplementary material. All new sequence data have been accessioned in GenBank: Accession numbers KU510405-416, KU527910-938.
