# Correction to ‘Young relicts and old relicts: a novel palaeoendemic vertebrate from the Australian Central Uplands’

**DOI:** 10.1098/rsos.161022

**Published:** 2017-01-18

**Authors:** Paul M. Oliver, Peter J. McDonald

## 1. Introduction

This correction is to fulfil the requirements of the International Commission on Zoological Nomenclature (ICZN) code criteria for the publication of new species name. In order for *Oedura luritja* to be a valid taxon, the species name needed to be registered in Zoobank at the time of publication, with the Zoobank number appearing with the publication. This correction aims to solve this issue, and the Zoobank LSID numbers are shown below along with a reiteration of the systematic section. The original work should be cited along with the correction when citing the new species.

## 2. Zoobank LSIDs

Original publication (Oliver & McDonald [1])

urn:lsid:zoobank.org:pub:746F48B7-41ED-4985-9604-B32B150FBE6E

Correction

urn:lsid:zoobank.org:pub:4FB222A3-FA9A-4D9A-A458-4EC826DD8B0E

New Species: *Oedura luritja*

urn:lsid:zoobank.org:act:548A4373-5656-4108-90BA-0014D6C29846

## 3. Systematics

*Oedura luritja* n. sp.

Mereenie velvet gecko

*Holotype.* NTM R37528, field number CCM5974, adult male with regrown tail, and liver samples stored in ethanol.

*Type locality.* Gorge 300 m east of north end of Boggy Hole (−24.13455, 132.86574), Finke Gorge National Park, Northern Territory, collected 5 October 2015 by P.J.M. and P.M.O.

*Paratypes.* All from Northern Territory (*n*  = 11). NTM R37529 (CCM5975) near Boggy Hole (−24.1351, 132.86351), Finke Gorge National Park, collected 5 October 2015; NTM R37531 (CCM5979) Palm Creek (−24.05449, 132.74246), Finke Gorge National Park; NTM R37530 (CCM5978) Palm Creek (−24.0584, 132.76151), Finke Gorge National Park, collected 6 October 2015; AMS R52143 Kings Canyon (−24.27, 131.57), Watarka National Park, collected 28 July 1975; AMS R52144–50 Reedy Springs (−24.30, 131.58), Watarka National Park, collected 28 July 1975.

*Etymology.* Luritja is a collective name for people speaking several dialects of the Aboriginal Western Desert language. The western parts of the distribution of *Oedura luritja* (including Watarrka National Park) are in Luritja lands. Luritja is also believed to be derived from the Arrernte word ‘Ulerenye’ meaning foreigner or stranger, and is therefore further appropriate for such a deeply divergent lineage. Used as a noun in apposition.

*Diagnosis.* A moderately large (to 99 mm SVL) species of *Oedura* with a moderately wide (HW/SVL 0.17–0.20) and flat head (HD/SVL 0.072–0.091), tail moderately long (original TL/SVL 0.65–0.87), narrow (TW/SVL 0.07–0.11), distinctly narrower than head and body and tapering gradually to a tip, rostral less than 50% divided, postcloacal spur usually single (22 out of 23 specimens), 10–16 precloacal pores in adult males, dorsal scales small (less than 0.5 mm in diameter), head brown with light flecking but with no trace of a light canthal stripe or dark-brown postorbital or nuchal stripes, and dorsal coloration of adults usually including five to six moderately well-defined light bands or transverse blotches (yellow in life) on a purplish brown background.
