# Supplementary material for: Young relicts and old relicts: a novel palaeoendemic vertebrate from the Australian Central Uplands
Source: R Soc Open Sci. 2016 Oct 5;3(10):160018. doi: 10.1098/rsos.160018 (PMC5098959; doi:10.1098/rsos.160018)
Supplement: 1. Supplementary methods. [file rsos160018supp1.docx]

**Supplementary methods**

**DNA extractions & mtDNA sequencing.**

DNA was extracted using the salting-out method of Sunnucks & Hales (1996). Sequences were amplified using the PCR primers given below. PCRs were performed in 25 μL reactions containing ~100 ng DNA, 2.5 μL 10X PCR Buffer, 0.5 mM dNTP, 1.5 mM MgCl2, 10 pmol each forward and reverse primer, 0.5 U Taq DNA Polymerase (Invitrogen). Cycling conditions included initial denaturation of 95 °C for 3 min, followed by a touchdown cycling of 94 °C for 30 s, 53–55 °C for 45 s, 68 °C for 45 s and a final extension of 68 °C for 3 min on a Corbett PC-960C cooled thermal cycler and included a negative control for each amplification.

PCR products were purified using 5 μL PCR product together with 0.4 lL Exonuclease 1 (New England BioLabs), 1.6 μL Shrimp Alkaline Phosphatase (In Vitro Technologies Pty. Ltd.) and 3 μL double-distilled water and incubated at 37 °C for 45 min followed by 80 °C for 15 min. Some PCR products were gel purified using Ultra-CleanTM15 DNA purification kit (MoBio Laboratories Inc.) following the manufacturer’s protocol.

Purified PCR products were sequenced in 20 μL reactions containing 1.0 μL BigDye Terminator v3.1 (Applied Biosystems), 4.5 μL 5X sequencing buffer, 3.2 pmol primer, 1 μL purified PCR product, 13.5 μL double-distilled water. Sequencing reactions contained 25 cycles of: denaturation of 94 °C for 5 s, annealing at 50 °C for 10 s and extension at 60 °C for 4 min. Products were purified using a sodium acetate cleanup method (see Pepper et al. 2006), eluted in 20 μL of HiDi formamide, then sequenced directly on an ABI 3100 DNA Analyzer (Applied Biosystems).

**Morphology.**

We recorded the following morphological data: snout-vent length (SVL), total length from tip of snout to the anterior edge of vent; head width (HW), maximum width of the head; head depth (HD), maximum depth of the head just posterior to the orbitals; head length (HL), from anterior edge of ear to tip of snout; eye to naris distance (EN), from anterior corner of eye to posterior edge of naris; internarial distance (IN), from inner edge of the nares; interorbital distance (IO), from the anterior-dorsal edge of the orbitals; transverse length of eye (EYE); axilla to groin (trunk) distance (Trk), from posterior edge of forelimb insertion to anterior edge of hindlimb insertion with limbs held at right angles; length of lower arm (ArmL) from posterior edge of bent elbow to wrist flexed at 90 degree; length of lower leg (LegL), from anterior edge of bent knee to heel flexed at 90 degrees; maximum width (including lamellae) of the 3^rd^ finger (3FW), and 3^rd^ toe (3TW), tail length (TL), and maximum tail width (TW) and depth (TD). Both original and regrown tails were measured, but they are reported as different ranges.

The following details of scalation were also recorded: number of supralabials (SuL), more than twice the size of surrounding granular scales (both total number and number to mid-point of eye); number of infralabials (InL), more than twice size of surrounding granular scales; number of small intervening scales along the dorsal edge of the nasals between the much larger lateral supranasals (InN); length of rostral crease (RC), from the dorsal edge of the rostral as a percentage of rostral height at the midpoint; total number of expressed precloacal pores (PP) on males; number of enlarged cloacal spurs (CS) on right and left side; and number of rows of enlarged lamellae (more than twice the width of surrounding scales) under the third finger (3FL) and third toe (3TL) (taken on left side only).

**Table S1** Details of primers used in this study.

| Primer pairs | Sequence | Reference | Fragment length |
| --- | --- | --- | --- |
| M112 F | AAGCTTTCGGGGCCCATACC | Sistrom *et al.* (2011) | *c.* 900 bp |
| M1123R | GCTTAATTAAAGTGTYTGAGTTGC | Sistrom *et al.* (2011) |  |
| PHO.F2 | AGA TGA GCA TGC AGG AGT ATG A | Bauer *et al.* (2007) | *c.* 450 bp |
| PHO.R1 | TCC ACA TCC ACA GCA AAA AAC TCC T | Bauer *et al.* (2007) |  |
| RAG1R13.F | TCT GAA TGG AAA TTC AAG CTG TT | Groth & Barrowclough (1999) | *c.* 1000 bp |
| RAG1R852.R | GAG TCT GCA GAA TAA GTG CTT GCA |  |  |
| RAG1R13.F | TCT GAA TGG AAA TTC AAG CTG TT | Groth & Barrowclough (1999) | *c.* 1000 bp |
| RAG1R18.R | GAT GCT GCC TCG GTC GGC CAC CTT T | Groth & Barrowclough (1999) |  |
| RAG1R13.F | TCT GAA TGG AAA TTC AAG CTG TT | Groth & Barrowclough (1999) | *c.* 800 bp |
| RAG1r.Stroph840 | AAG TGC TTG CAT GTT GTT TC | Nielson et al. (2016) |  |

References

Bauer, A.M., de Silva, A., Greenbaum, E. & Jackman, T. (2007) A new species of day gecko from high elevation in Sri Lanka, with a preliminary phylogeny of Sri Lanka *Cnemaspis* (Reptilia, Squamata, Gekkonidae). *Mitteilungen aus dem Museum für Naturkunde in Berlin, Zoologische Reihe*, **83** (S1), 22–32.

Groth, J.G. & Barrowclough, G.F. (1999) Basal divergences in birds and the phylogenetic utility of the nuclear RAG-1 gene. *Molecular Phylogenetics and Evolution*, **12**, 115–123.

Nielsen, S.V., Oliver, P.M., Laver, R., Bauer, A.M., Noonan, B.P. Stripes, Jewels and Spines: further investigations of the evolution of defensive strategies in a chemically defended gecko radiation (*Strophurus*, Diplodactylidae). *Zoologica Scripta.* Avaliable online. DOI. 10.1111/zsc.12181

Sistrom M, Hutchinson M, Hutchinson R, & Donnellan S (2009). Molecular phylogeny of Australian Gehyra (Squamata: Gekkonidae) and taxonomic revision of *Gehyra variegata* in south-eastern Australia. Zootaxa, 2277, 14-32

Sunnucks P, Hales DF (1996) Numerous transposed sequences of mitochondrial cytochrome oxidase I-II in aphids of the genus Sitobion (Hemiptera: Aphididae). *Molecular Biology & Evolution*, 13, 510–524.
