# Supplementary material for: Young relicts and old relicts: a novel palaeoendemic vertebrate from the Australian Central Uplands
Source: R Soc Open Sci. 2016 Oct 5;3(10):160018. doi: 10.1098/rsos.160018 (PMC5098959; doi:10.1098/rsos.160018)
Supplement: 2. Supplementary Tables and Appendices: Table S1. Specimens number, locality data and genbank numbers for all samples included in genetic analyses; Table S2. Summary of results from dating analyses. Table S3; Summary of morphological data for velvet geckos in the genus Oedura from the Australian Ari [file rsos160018supp2.docx]

Supplementary Tables and Appendices.

Table S1. Summary information for samples included in genetic analyses including both *Oedura* and outgroups.

| **Taxon** | **REGNO** | **Tissue number** | **RAG-1** | **Phos** | **ND2** | **LOCALITY** | **State** | **LAT** | **LONG** |
| --- | --- | --- | --- | --- | --- | --- | --- | --- | --- |
| *Oedura bella* | NTMR21288 |  | KJ803670 | KJ803711 | KJ803591 | Musselbrook Reservoir | QLD | -18.4000 | 138.3133 |
| *Oedura bella* | SAMAR34188 |  | KJ803671 | KJ803712 | KJ803592 | McArthur R. Stn. | NT | -16.6667 | 135.8500 |
| *Oedura bella* | SAMAR34209 |  |  |  | KJ803593 | Lawn Hill NP | Qld | -18.5830 | 138.5000 |
| *Oedura bella* | SAMAR35425 |  | KJ803672 | KJ803713 | KJ803594 | Mt. Isa | QLD | -20.7333 | 139.4833 |
| *Oedura cincta* Central | AMSR136049 |  | JQ173738 | JQ173690 | JQ173644 | Vicinity of 8 Mile Bore, Tanami Road | NT | -22.1160 | 131.366 |
| *Oedura cincta* Central | AMSR178154 | EBU81451 |  |  | KU527936 | W of Alice Springs, Namatjira Dr, 28.0km W of Larapinta Dr intersection | NT | -23.80974 | 133.20647 |
| *Oedura cincta* Central | AMSR178155 | EBU81452 |  |  | KU527937 | W of Alice Springs, Namatjira Dr, 28.0km W of Larapinta Dr intersection | NT | -23.80974 | 133.20647 |
| *Oedura cincta* Central | AMSR178167 | EBU81464 |  |  | KU527935 | W of Alice Springs, Namatjira Dr, 28.0km W of Larapinta Dr intersection | NT | -23.80974 | 133.20647 |
| *Oedura cincta* Central | AMSR180744 | EBU31384 |  |  | KU527938 | 18km along Ross River road from Stuart Hwy. | NT | -23.75 | 133.02 |
| *Oedura cincta* Central | CCM5968 |  |  |  | KU527933 | Lawrence Gorge | NT | -24.006582 | 133.436173 |
| *Oedura cincta* Central | CCM5969 |  |  |  | KU527934 | Lawrence Gorge | NT | -24.006582 | 133.436173 |
| *Oedura cincta* Central | CCM5998 | CCM5998 |  |  | KU527930 | Roma Gorge | NT | -23.65595 | 132.41298 |
| *Oedura cincta* Central | CCM6003 |  |  |  | KU527931 | Roma Gorge | NT | -23.65595 | 132.41298 |
| *Oedura cincta* Central | CCM6004 |  |  |  | KU527932 | Roma Gorge | NT | -23.65595 | 132.41298 |
| *Oedura cincta* Central | NTMR18278 |  | KJ803662 | KJ803703 | KJ803565 | Paddy's Rockhole | NT | -35.7608 | 137.9950 |
| *Oedura cincta* Central | NTMR35902 |  |  |  | KJ803566 | 10km S of Mt Doreen ruins | NT | -22.1062 | 131.3857 |
| *Oedura cincta* Central | NTMR35912 |  |  |  | KJ803567 | 10km S of Mt Doreen ruins | NT | -22.1062 | 131.3857 |
| *Oedura cincta* Central | SAMAR38842 |  | JQ173742 | JQ173694 | JQ173648 | Honeymoon Gap, Alice Springs | NT | -23.7500 | 133.7500 |
| *Oedura cincta* Central | SAMAR38843 |  |  |  | KJ803568 | Honeymoon Gap, Alice Springs | NT | -23.7500 | 133.7500 |
| *Oedura cincta* Central | SAMAR38844 |  |  |  | KJ803569 | 3k W Stanley Chasm t/off | NT | -23.7667 | 133.5333 |
| *Oedura cincta* Central | SAMAR38845 |  |  |  | KJ803570 | 3k W Stanley Chasm t/off | NT | -23.7667 | 133.5333 |
| *Oedura cincta* Central | SAMAR65905 |  |  |  | KJ803571 | Emily Gap | NT | -23.7397 | 133.9506 |
| *Oedura cincta* Eastern | AMSR138446 |  | JQ173739 | JQ173691 | JQ173645 | Bourke, west of Warego River Bridge | NSW | -30.0000 | 145.3500 |
| *Oedura cincta* Eastern | No voucher | ABTC130284 |  |  | KU527923 | 5.4k W Lochern Homestead, Lochern NP | Qld | -24.11568 | 143.34984 |
| *Oedura cincta* Eastern | No voucher | ABTC130285 |  |  | KU527922 | 5.4k W Lochern Homestead, Lochern NP | Qld | -24.11568 | 143.34984 |
| *Oedura cincta* Eastern | No voucher | ABTC127625 |  |  | KU527927 | Quilpie - Thargominda Road, near Quilpie | Qld | -26.88 | 144.19 |
| *Oedura cincta* Eastern | No voucher | ABTC130276 |  |  | KU527921 | Idalia Homestead, Idalia NP | Qld | -24.88347 | 144.77454 |
| *Oedura cincta* Eastern | No voucher | ABTC124396 |  |  | KU527929 | 60k W Cobar, Meadow Glen Rest Stop | NSW | -31.54 | 145.23 |
| *Oedura cincta* Eastern | SAMAR41324 |  | KJ803663 | KJ803704 | KJ803573 | Oakbank Station | SA | -33.1278 | 140.6444 |
| *Oedura cincta* Eastern | SAMAR42883 |  | KJ803664 | KJ803705 | KJ803574 | 30 KM Eof Noonbah Station | QLD | -24.1167 | 143.4167 |
| *Oedura cincta* Eastern | SAMAR42913 |  |  |  | KJ803575 | 85 km W Windorah | QLD | -25.3500 | 141.8333 |
| *Oedura cincta* Eastern | SAMAR52203 |  | JQ173743 | JQ173695 | JQ173649 | 2.7k W Lance Bore Narrina Stn | SA | -30.9567 | 138.7730 |
| *Oedura cincta* Eastern | SAMAR52333 |  |  |  | KJ803576 | 6k ESE Blackwater Springs,Oratunga | SA | -31.0972 | 138.8836 |
| *Oedura cincta* Eastern | SAMAR54300 |  | KJ803665 | KJ803706 | KJ803577 | W Of Bellbird Campsite, Gluepot Reserve | SA | -33.7028 | 140.1631 |
| *Oedura cincta* Eastern | SAMAR55902 | ABTC79483 |  |  | KU527926 | 9k N New South Wales/Queensland border on Mitchell Highway | Qld | -28.960278 | 145.727778 |
| *Oedura cincta* Eastern | SAMAR55903 | ABTC79484 |  |  | KU527928 | 9k N New South Wales/Queensland border on Mitchell Highway | Qld | -28.960278 | 145.727778 |
| *Oedura cincta* Eastern | ABTC113886 |  |  |  | KJ803572 | 0.5k W Ballards tank, Plevna Downs | Qld | -26.7394 | 142.5628 |
| *Oedura cincta* Eastern | ABTC113859 | ABTC113859 |  |  | KU527925 | Noonbah dam | Qld | -24.103889 | 143.185556 |
| *Oedura cincta* Eastern | ABTC113860 | ABTC113860 |  |  | KU527924 | 6.7k E Noonbah Homestead | Qld | -24.115278 | 143.251667 |
| *Oedura filicipoda* | AMSR126183 |  | JQ173730 | JQ173682 | JQ173636 | Little Mertens Falls | WA | -14.8160 | 125.7160 |
| *Oedura filicipoda* | WAMR138874 |  | KJ803729 | KJ803729 | KJ803581 | Donkin's Hill, Mitchell Plateau | WA | -14.9875 | 125.5069 |
| *Oedura filicipoda* | WAMR167805 |  |  |  | KJ803579 | Mitchell Plateau | WA | -14.6733 | 125.7322 |
| *Oedura filicipoda* | WAMR171552 |  |  |  | KJ803580 | Prince Regent Nature Reserve | WA | -15.7613 | 125.2575 |
| *Oedura fimbria* | WAMR102618 |  |  |  | KJ803622 | Little Sandy Desert | WA | -24.0536 | 120.4067 |
| *Oedura fimbria* | WAMR102619 |  |  |  | KJ803623 | Little Sandy Desert | WA | -24.0536 | 120.4067 |
| *Oedura fimbria* | WAMR102622 |  |  |  | KJ803624 | Little Sandy Desert | WA | -24.0536 | 120.4067 |
| *Oedura fimbria* | WAMR105965 |  | JQ173744 | JQ173696 | JQ173650 | 7k N Mt Magnet WA | WA | -28.0097 | 117.8494 |
| *Oedura fimbria* | WAMR106289 |  |  |  | KJ803625 | 7KM N Mount Magnet | WA | -28.0000 | 117.8500 |
| *Oedura fimbria* | WAMR110173 |  |  |  | KJ803626 | 4KM SW Hugh Bluff | WA | -22.0592 | 117.6930 |
| *Oedura fimbria* | WAMR111891 |  |  |  | KJ803627 | Balfour Downs | WA | -23.5000 | 120.1000 |
| *Oedura fimbria* | WAMR114252 |  |  |  | KJ803628 | NW end of Mount Fraser | WA | -25.5833 | 118.3667 |
| *Oedura fimbria* | WAMR119086 |  |  |  | KJ803629 | Virgin Springs, Carnarvon Range | WA | -25.1000 | 120.7167 |
| *Oedura fimbria* | WAMR119837 |  |  |  | KJ803630 | Yandicoogina WA | WA | -21.3997 | 120.1800 |
| *Oedura fimbria* | WAMR119991 |  |  |  | KJ803631 | Hope Downs | WA | -23.0083 | 119.1028 |
| *Oedura fimbria* | WAMR119992 |  |  |  | KJ803632 | Mount Brockman | WA | -22.4666 | 117.3000 |
| *Oedura fimbria* | WAMR119993 |  |  |  | KJ803633 | Hope Downs | WA | -23.0000 | 119.1167 |
| *Oedura fimbria* | WAMR125106 |  |  |  | KJ803634 | Yandicoogina | WA | -22.7166 | 119.0167 |
| *Oedura fimbria* | WAMR127833 |  |  |  | KJ803635 | Mount Brockman, Namuldin | WA | -22.3105 | 117.3219 |
| *Oedura fimbria* | WAMR129595 |  |  |  | KJ803636 | 120KM NW Newman | WA | -22.9166 | 118.8833 |
| *Oedura fimbria* | WAMR129622 |  |  |  | KJ803637 | 120KM NW Newman | WA | -22.9166 | 118.8833 |
| *Oedura fimbria* | WAMR129635 |  |  |  | KJ803638 | 120KM NW Newman | WA | -22.9166 | 119.0167 |
| *Oedura fimbria* | WAMR132296 |  |  |  | KJ803639 | Ulongunna Rock | WA | -27.1194 | 117.2331 |
| *Oedura fimbria* | WAMR132626 |  | KJ803724 | KJ803724 | KJ803640 | Burrup Peninsula | WA | -20.5963 | 116.8108 |
| *Oedura fimbria* | WAMR132633 |  |  |  | KJ803642 | Burrup Peninsula | WA | -20.5963 | 116.8108 |
| *Oedura fimbria* | WAMR135369 |  |  |  | KJ803643 | Mount Brockman | WA | -22.3105 | 117.3219 |
| *Oedura fimbria* | WAMR135445 |  |  |  | KJ803644 | Mount Brockman | WA | -22.3105 | 117.3219 |
| *Oedura fimbria* | WAMR135447 |  |  |  | KJ803645 | Mount Brockman | WA | -22.3105 | 117.3219 |
| *Oedura fimbria* | WAMR135448 |  |  |  | KJ803646 | Mount Brockman | WA | -22.3105 | 117.3219 |
| *Oedura fimbria* | WAMR135449 |  |  |  | KJ803647 | Mount Brockman | WA | -22.3105 | 117.3219 |
| *Oedura fimbria* | WAMR146593 |  |  |  | KJ803648 | 40km SE Pouyouwuncubban | WA | -22.1494 | 119.0186 |
| *Oedura fimbria* | WAMR146594 |  |  |  | KJ803649 | 40km SE Pouyouwuncubban | WA | -22.1494 | 119.0186 |
| *Oedura fimbria* | WAMR154783 |  | KJ803725 | KJ803725 | KJ803650 | Brockman Ridge | WA | -23.3091 | 119.8861 |
| *Oedura fimbria* | WAMR154785 |  |  |  | KJ803651 | Brockman Ridge | WA | -23.3108 | 119.9169 |
| *Oedura fimbria* | WAMR154796 |  |  |  | KJ803652 | Walga Rock | WA | -27.3986 | 117.4708 |
| *Oedura fimbria* | WAMR154797 |  | KJ803726 | KJ803726 | KJ803653 | Walga Rock | WA | -27.3986 | 117.4708 |
| *Oedura fimbria* | WAMR157504 |  |  |  | KJ803654 | Packsaddle Range | WA | -22.9169 | 118.8900 |
| *Oedura fimbria* | WAMR157516 |  |  |  | KJ803655 | Packsaddle Range | WA | -22.9169 | 118.8900 |
| *Oedura fimbria* | WAMR157595 |  |  |  | KJ803656 | West Angelas | WA | -23.1869 | 118.8606 |
| *Oedura fimbria* | WAMR160066 |  |  |  | KJ803657 | 58KM ESE Meentheena Outcamp | WA | -21.3219 | 121.0020 |
| *Oedura fimbria* | WAMR160074 |  | KJ803727 | KJ803727 | KJ803658 | 32.5KM ESE Meentheena Outcamp | WA | -21.3337 | 120.7520 |
| *Oedura fimbria* | WAMR165150 |  |  |  | KJ803659 | 1.5KM NNW Python Pool | WA | -21.3210 | 117.2310 |
| *Oedura fimbria* | WAMR165241 |  |  |  | KJ803660 | Hanson Cove | WA | -20.6352 | 116.7989 |
| *Oedura fimbria* | WAMR165242 |  | KJ803728 | KJ803728 | KJ803661 | Burrup Peninsula, Hearson Cove | WA | -20.6350 | 116.7990 |
| *Oedura gemmata* | NMVD72584 | NMVD72584 |  |  | KJ803582 | Near Kikiyown (also called "Kikikyon") | NT | -12.1883 | 133.8125 |
| *Oedura gemmata* | NMVD72598 | NMVD72598 |  |  | KJ803583 | Near Kikiyown (also called "Kikikyon") | NT | -12.1883 | 133.8125 |
| *Oedura gemmata* | NMVD72599 | NMVD72599 |  |  | KJ803584 | Near Kikiyown (also called "Kikikyon") | NT | -12.1883 | 133.8125 |
| *Oedura gemmata* | NMVD72600 | NMVD72600 |  |  | KJ803585 | Near Kikiyown (also called "Kikikyon") | NT | -12.1883 | 133.8125 |
| *Oedura gemmata* | NMVD72621 | NMVD72621 |  |  | KJ803586 | Near Kikiyown (also called "Kikikyon") | NT | -12.1883 | 133.8125 |
| *Oedura gemmata* | NTMR34986 | NTMR34986 | KJ803708 | KJ803708 | KJ803587 | Mountain Valley Station | NT | -13.9805 | 133.1772 |
| *Oedura gemmata* | NTMR34987 | NTMR34987 | KJ803709 | KJ803709 | KJ803588 | Mountain Valley Station | NT | -13.9805 | 133.1772 |
| *Oedura gemmata* | NTMR35680 | NTMR35680 | KJ803710 | KJ803710 | KJ803589 | Kakadu National Park | NT | -13.8617 | 132.9788 |
| *Oedura gemmata* | NTMR35753 | NTMR35753 |  |  | KJ803590 | Oenpelli, Reservoir | NT | -12.3762 | 133.0722 |
| *Oedura gemmata* | SAMAR34170 |  | JQ173731 | JQ173683 | JQ173637 | UDP Falls | NT | -13.4333 | 132.4167 |
| *Oedura gracilis* | AMSR136067 | AMSR136067 | JQ173732 | JQ173684 | JQ173638 | Bells Gorge, Bells Crk, Isdell River. | WA | -16.9830 | 125.1830 |
| *Oedura gracilis* | AMSR140309 | AMSR140309 | JQ173733 | JQ173685 | JQ173639 | Manning Gorge, Mt. Barnett Station | WA | -16.6586 | 125.9269 |
| *Oedura gracilis* | NTMR13438 | NTMR13438 | KJ803741 | KJ803741 | KJ803620 | Bulloo River Station | NT | -15.4667 | 129.7500 |
| *Oedura gracilis* | WAMR138891 | WAMR138891 | KJ803738 | KJ803738 | KJ803617 | Donkin's Hill, Mitchell Plateau | WA | -14.9875 | 125.5069 |
| *Oedura gracilis* | WAMR151005 | WAMR151005 | KJ803740 | KJ803740 | KJ803619 | Warmun | WA | -16.7508 | 128.2867 |
| *Oedura gracilis* | WAMR151963 | WAMR151963 | KJ803742 | KJ803742 | KJ803621 | South West Osborn Island | WA | -14.3500 | 125.9500 |
| *Oedura gracilis* | WAMR156724 | WAMR156724 | KJ803730 | KJ803730 | KJ803609 | Piccaninny Massif | WA | -17.4002 | 128.4117 |
| *Oedura gracilis* | WAMR156728 | WAMR156728 | KJ803731 | KJ803731 | KJ803610 | Tunnel Creek | WA | -17.6377 | 125.1689 |
| *Oedura gracilis* | WAMR164910 | WAMR164910 | KJ803732 | KJ803732 | KJ803611 | Katers Island | WA | -14.4500 | 125.5208 |
| *Oedura gracilis* | WAMR168564 | WAMR168564 | KJ803733 | KJ803733 | KJ803612 | Boongaree Island | WA | -15.1000 | 125.2000 |
| *Oedura gracilis* | WAMR168565 | WAMR168565 | KJ803734 | KJ803734 | KJ803613 | Augustus Island | WA | -15.3500 | 124.5333 |
| *Oedura gracilis* | WAMR171668 | WAMR171668 | KJ803735 | KJ803735 | KJ803614 | Storr Island | WA | -15.9500 | 124.5611 |
| *Oedura gracilis* | WAMR171670 | WAMR171670 | KJ803736 | KJ803736 | KJ803615 | Lachlan Island | WA | -16.6236 | 123.4714 |
| *Oedura gracilis* | WAMR172341 | WAMR172341 | KJ803737 | KJ803737 | KJ803616 | Theda Station | WA | 14.8000 | 126.5000 |
| *Oedura gracilis* | WAMR172865 | WAMR172865 | KJ803739 | KJ803739 | KJ803618 | Ellenbrae | WA | -15.9839 | 127.0539 |
| *Oedura luritja* |  | CCM5973 | KU510412 | KU510406 | KU527915 | Finke Gorge National Park, small gorge just east of Boggy Hole | NT | -24.13455 | 132.86574 |
| *Oedura luritja* |  | CCM5974 |  |  | KU527914 | Finke Gorge National Park, small gorge just east of Boggy Hole | NT | -24.13455 | 132.86574 |
| *Oedura luritja* |  | CCM5975 |  |  | KU527916 | Finke Gorge National Park, small gorge just east of Boggy Hole | NT | -24.1351 | 132.86351 |
| *Oedura luritja* |  | CCM5976 |  |  | KU527917 | Finke Gorge National Park, behind old ranger station | NT | -24.0584 | 132.76151 |
| *Oedura luritja* |  | CCM5977 |  |  | KU527918 | Finke Gorge National Park, behind old ranger station | NT | -24.05449 | 132.74246 |
| *Oedura luritja* |  | CCM5978 | KU510411 | KU510405 | KU527920 | Finke Gorge National Park, behind old ranger station | NT | -24.0584 | 132.76151 |
| *Oedura luritja* |  | CCM5979 | KU510413 | KU510407 | KU527919 | Finke Gorge National Park, near palm creek | NT | -24.05449 | 132.74246 |
| *Oedura luritja* |  | CCM6227 | KU510414 | KU510408 | KU527910 | Kings Canyon, George Gill Range | NT | -24.25576 | 131.57649 |
| *Oedura luritja* |  | CCM6228 | KU510415 | KU510409 | KU527911 | Kings Canyon, George Gill Range | NT | -24.24966 | 131.57366 |
| *Oedura luritja* |  | CCM6229 | KU510416 | KU510410 | KU527912 | Kathleen Springs, George Gill Range | NT | -24.33941 | 131.68045 |
| *Oedura luritja* | SAMAR65915 | ABTC112658 |  |  | KU527913 | Rainbow Valley, 11.7k E Mt Grevillea | NT | -24.333889 | 133.635278 |
| *Oedura marmorata* North 4 | NTMR22444 |  | KJ803682 | KJ803723 | KJ803608 | Limmen Gate NP | NT | -15.7806 | 135.3311 |
| *Oedura marmorata* North1 | NTMR13222? |  | KJ803673 | KJ803714 | KJ803596 | Urapunga Station | NT | -14.5100 | 134.5700 |
| *Oedura marmorata* North1 | NTMR13295 |  | KJ803674 | KJ803715 | KJ803597 | Victoria R. Gregory NP. | NT | -15.9433 | 130.5006 |
| *Oedura marmorata* North1 | NTMR13619 |  | JQ173741 | JQ173693 | JQ173647 | 3km S Katherine | NT | -14.47 | 132.27 |
| *Oedura marmorata* North1 | NTMR19029 |  | KJ803675 | KJ803716 | KJ803598 | Marchinbar Is, (North) | NT | -11.1978 | 136.6986 |
| *Oedura marmorata* North1 | NTMR19030 |  | KJ803676 | KJ803717 | KJ803599 | Marchinbar Is, (North) | NT | -11.1978 | 136.6986 |
| *Oedura marmorata* North1 | NTMR22774 |  | KJ803677 | KJ803718 | KJ803600 | English Company Isles, Astell Is | NT | -11.8797 | 136.4222 |
| *Oedura marmorata* North1 | NTMR36709 |  |  |  | KJ803595 | Fish River | NT | xxx | xxx |
| *Oedura marmorata* North1 | NTMR36717 |  |  |  | KJ803601 | Wongalarra | NT | -14.1780 | 134.3640 |
| *Oedura marmorata* North1 | NTMR36731 |  |  |  | KJ803602 | Wongalarra | NT | -14.1529 | 134.1611 |
| *Oedura marmorata* North1 | NTMR36746 |  | KJ803678 | KJ803719 | KJ803603 | Wongalarra | NT | -14.1328 | 134.3430 |
| *Oedura marmorata* North2 | SAMAR34156 |  | KJ803679 | KJ803720 | KJ803604 | 1km N Katherine | NT | -14.4583 | 132.2583 |
| *Oedura marmorata* North2 | SAMAR34158 |  | KJ803680 | KJ803721 | KJ803605 | 1km N Katherine | NT | -14.4583 | 132.2583 |
| *Oedura marmorata* North3 | NTMR22104 |  | KJ803681 | KJ803722 | KJ803606 | Cox Peninsula Rd | NT | -12.5167 | 130.8167 |
| *Oedura marmorata* North3 | NTMR22183 |  |  |  | KJ803607 | Litchfield NP | NT | -13.4086 | 130.8967 |
| *Oedura murrumanu* |  | PMO177 |  |  | KU522241 | Oscar Range | WA | -17.67509 | 125.07004 |
| *Oedura murrumanu* |  | PMO185 |  |  | KU522239 | Oscar Range | WA | -17.6748 | 125.0704 |
| *Oedura murrumanu* |  | PMO190 |  |  | KU522240 | Oscar Range | WA | -17.67512 | 125.07004 |
| *Oedura murrumanu* | NMVD77002 | PMO51 |  |  | KM016836 | Oscar Range | WA | -17.9125 | 125.2827 |
| *Oedura murrumanu* | WAMR173369 | PMO52 |  |  | KM016837 | Oscar Range | WA | -17.9125 | 125.2827 |
| *Oedura murrumanu* | NMV76947 | PMO53 |  |  | KM016838 | Oscar Range | WA | -17.9125 | 125.2827 |
| *Oedura murrumanu* | WAMR173368 | PMO54 |  |  | KM016839 | Oscar Range | WA | -17.9166 | 125.3024 |
| *Oedura murrumanu* | NMV76948 | PMO55 |  |  | KM016840 | Oscar Range | WA | -17.9166 | 125.3024 |
| *Oedura murrumanu* | WAMR173370 | PMO56 |  |  | KM016841 | Oscar Range | WA | -17.9166 | 125.3024 |
| **Outgroups** |  |  |  |  |  |  |  |  |  |
|  |  |  |  | GenBank Accession numbers | |  |  |  |  |
| Species | Museum Number |  | *RAG-1* | *PDC* | *ND2* | Locality |  |  |  |
|  |  |  | AY662627 | _ | AY369016 |  |  |  |  |
| *Crenadactylus ocellatus* | SAMA R22245 |  | JQ173721 | JQ173673 | JQ173628 | NT: 10k S Barrow Ck |  |  |  |
| *Diplodactylus conspicillatus* | AMS R158426 |  | JQ173722 | JQ173674 | JQ173628 | NSW: Sturt National Park |  |  |  |
| *Diplodactylus granariensis* | AMS R150637 |  | JQ173723 | JQ173675 | JQ173629 | WA: Dedari |  |  |  |
| *Diplodactylus ornatus* | AMS R140546 |  | JQ173725 | JQ173677 | JQ173631 | WA: Denham |  |  |  |
| *Diplodactylus tessellatus* | AMS R143855 |  | JX024503 | **_** | JX024362 | QLD: 7.9km Sw of Landborough Hwy on Boulia Rd | |  |  |
| *Lucasium maini* | AMS150647 |  | JQ173724 | JQ173676 | JQ173630 | WA |  |  |  |
| *Lucasium stenodactylum* | AMS R139897 |  | GU459409 | GU459611 | GU459812 | WA: El Questro Station |  |  |  |
| *Mokopirirakau granulatus* | RAH363 |  | JQ173726 | JQ173678 | JQ173632 | NZ: Trass |  |  |  |
| *Oedodera marmorata* | AMS R161254 |  | JQ173727 | JQ173679 | JQ173633 | NC: Sommet Noir, Paagoumène, 11 km NW Koumac | |  |  |
| *Oedura castelnaui* | AMS R143917 |  | JQ173728 | JQ173680 | JQ173634 | QLD: 4.9km E Georgetown |  |  |  |
| *Oedura castelnaui* | SAMA R55715 |  | JQ173729 | JQ173681 | JQ173635 | QLD: Kennedy Rd turnoff to Porcupine Gorge |  |  |  |
| *Oedura coggeri* | AMS R143918 |  | JQ173734 | JQ173686 | JQ173640 | QLD: 20.0km W Gulf Development Rd & Kennedy Hwy |  |  |  |
| *Amalosia lesueurii* | AMS R152230 |  | JQ173736 | JQ173688 | JQ173642 | NSW: Marramarra National Park |  |  |  |
| *Amalosia lesueurii* | AMS R159546 |  | JQ173737 | JQ173689 | JQ173643 | NSW: Moonbi Lookout, Moonbi Ranges |  |  |  |
| *Amalosia lesueurii* | AMS R161008 |  | JQ173746 | JQ173698 | JQ173652 | NSW: Arakoola Nature Reserve |  |  |  |
| *Oedura monilis* | SAMA R54507 |  | JQ173747 | JQ173699 | JQ173653 | QLD: 27k N Porcupine Ck campground. |  |  |  |
| *Oedura monilis* | SAMA R54560 |  | JQ173745 | JQ173697 | JQ173651 | QLD: Dawson Development Rd, 18k E Alpha T/off |  |  |  |
| *Oedura monilis* | AMS R152056 |  | JQ173748 | JQ173701 | JQ173655 | NSW: Warrumbungle National Park, Gould's circuit |  |  |  |
| *Amalosia obscura* | AMS R136124 |  |  | JQ173700 | JQ173654 | WA: 4km NE of Surveyors Pool, Mitchell Plateau |  |  |  |
| *Amalosia obscura* | AMS R136077 |  | FJ855450 | JQ173703 | EF681803 | WA: 3.5km upstream from Bells Gorge, Isdell River. |  |  |  |
| *Hesperoedura reticulata* | SAMA R23035 |  | JQ173755 | JQ173709 | JQ173661 | WA: 73k E Norseman |  |  |  |
| *Amalosia rhombifer* | SAMA R55604 |  | JQ173753 | JQ173707 | JQ173659 | QLD: Kroombit Tops |  |  |  |
| *Amalosia rhombifer* | NTMR 22222 |  | JQ173754 | JQ173708 | JQ173660 | NT: Litchfeild National Park |  |  |  |
| *Amalosia rhombifer* | SAMA R34513 |  | JQ173751 | JQ173705 | JQ173657 | QLD: Townsville |  |  |  |
| *Amalosia rhombifer* | AMS R140413 |  | JQ173752 | JQ173706 | JQ173658 | WA: Roebuck Bay, Broome Bird Observatory |  |  |  |
| *Amalosia rhombifer* | AMS R142587 |  | JQ173756 | JQ173710 | JQ173662 | QLD: Lamb Range |  |  |  |
| *Nebulifera robusta* | ABTC 3938 (tissue) |  | JQ173757 | JQ173711 | JQ173663 | QLD: near Rathdowney |  |  |  |
| *Oedura tryoni* | AMS R152045 |  | JQ173758 | JQ173712 | JQ173664 | NSW: Moonbi Lookout, Moonbi Ranges |  |  |  |
| *Oedura tryoni* | AMS R157247 |  | HQ288425 | _ | FJ855449 | NSW: 19.2 km W Tenterfield |  |  |  |
| *Pseudothecadactylus australis* | QMJ R57120 |  | AY662626 | _ | AY369024 | QLD: Heathlands |  |  |  |
| *Pseudothecadactlus lindneri* | AMS R90915 |  | JQ173759 | JQ173713 | JQ173665 | NT: Liverpool R |  |  |  |
| *Rhacodactylus chahoua* | AMS R161238 |  | GU459553 | GU459755 | GU459954 | NC: Dome de Tiebaghi, 14 km NW Koumac |  |  |  |
| *Rhynchoedura ornata* | AMS R155371 |  | JQ173760 | JQ173714 | JQ173666 | NSW: Sturt National Park |  |  |  |
| *Strophurus assimilis* | AMS R149832 |  | JQ173761 | JQ173715 | JQ173667 | WA:17.6 W Bonny Vale Railway Station |  |  |  |
| *Strophurus ciliaris aberrans* | AMS R136023 |  | JQ173762 | JQ173716 | JQ173668 | WA: Tanami Hwy |  |  |  |
| *Strophurus ciliaris ciliaris* | AMS R147216 |  | JQ173763 | JQ173717 | JQ173669 | NT: Barkley Hwy |  |  |  |
| *Strophurus elderi* | AMS R130987 |  | GU459551 | GU459753 | GU459952 | NSW: 17,9km N of Coombah Roadhouse |  |  |  |
| *Strophurus intermedius* | AMS R158434 |  | JQ173764 | JQ173718 | JQ173670 | NSW: 35 km from Mt Hope |  |  |  |
| *Strophurus rankini* | AMS R140490 |  | JQ173765 | JQ173719 | JQ173671 | WA: Coral Bay |  |  |  |
| *Strophurus spinigerus* | AMS R149815 |  | JQ173766 | JQ173720 | JQ173672 | WA: Buckland Hill |  |  |  |
| *Strophurus strophurus* | AMS R140536 |  | GU459449 | GU459651 | GU459852 | WA: Denham |  |  |  |

Table S2. Prior and posterior mean ages estimates and 95% Posterior distribution of age estimates for key clades (in millions of years before present). Age estimates respectively: nuc uncln = nuclear alignment only with uncorrelated lognormal; nuc strict = nuclear aligment only with strict clock; comb ucln = nuclear plus mitochondrial 1^st^ and 2^nd^ codons (thirds excluded) and uncorrelated lognormal, and; comb strict = nuclear plus mitochondrial 1^st^ and 2^nd^ codons (thirds excluded) and strict clock. All estimates made using BEAST version 1.8.

|  |  |  |  |  |
| --- | --- | --- | --- | --- |
|  | nuc ucln | nuc strict | comb ucln | comb strict |
| *Priors* |  |  |  |  |
| Australia-New Caledonia | 42 (9) | 42 (9) | 42 (9) | 42 (9) |
| Core Diplodactylidae | 35 (6) | 35 (6) | 35 (6) | 35 (6) |
| *Posterior crown ages* |  |  |  |  |
| Australia-New Caledonia | 37.6 (24.0-50.8) | 37.7 (26.8-51.3) | 36.8 (23.2-52.3) | 42.0 (28.2-55.5) |
| Core Diplodactylidae | 31.3 (19.7-38.0) | 30.1 (20.4-38.1) | 33.0 (23.1-44.3) | 30.0 (21.0-39.8) |
| All *Oedura* | 14.5 (8.5.3-20.7) | 12.8 (8.9-19.2) | 25.1 (16.9-34.4) | 21.3 (14.3-28.2) |
| ((*cincta*) (*fimbria*)) | 6.3 (3.4-11.4) | 5.8 (2.8-8.3) | 11.1 (6.3-16.5) | 9.6 (6.1-13.7) |
| *cincta* | 4.6 (2.0-7.7) | 4.3 (2.1-6.8) | 6.0 (3.0-9.6) | 5.6 (3.2-8.2) |
| *cincta* Central | 2.0 (0.3-4.5) | 2.0 (0.5-4.0) | 3.7 (1.4-6.5) | 3.5 (1.9-5.6) |
| ***luritja* n. sp. v other *Oedura*** | **12.8 (7.1-19.2)** | **11.6 (8.1-15.9)** | **23.8 (15.9-32.6)** | **20.2 (14.1-27.6)** |

Table S3. Summary of morphological data for *Oedura* from the Australian arid zone. Details of measurement acronyms are given in supplementary methods. Values are respectively mean, standard deviation and min and max in parentheses.

|  | *Oedura luritja* **sp. nov.** | *Oedura cincta* (eastern) | *Oedura cincta* (central) | *Oedura fimbria* |
| --- | --- | --- | --- | --- |
|  | n = 18 | n = 32 | n = 23 | n = 28 |
| SVL | 93.4±3.9 (85.0–99.0) | 90.6±6.0 (78.0–100.0) | 95.5±7.2 (82.0–108.0) | 94.3±6.7 (71.0–104.0) |
| HW | 17.4±1.2 (15.2–19.0) | 16.3±1.1 (14.1–18.5) | 17.7±1.1 (15.9–20.0) | 18.1±1.3 (14.3–20.3) |
| HD | 7.7±0.5 (6.9–8.5) | 9.6±0.6 (8.3–10.5) | 9.1±0.7 (7.6–10.1) | 9.6±0.9 (7.2–11.3) |
| HL | 21.9±0.9 (20.1–23.2) | 20.5±1.3 (18.5–22.9) | 22.3±1.2 (19.7–24.3) | 23.3±1.3 (18.6–24.9) |
| SuL | 10.8±0.9 (10.0–12.0) | 10.4±0.8 (9.0–13.0) | 9.7±0.6 (9.0–11.0) | 11.1±0.6 (9.0–14.0) |
| InL | 12.7±1.0 (11.0–15.0) | 11.2±0.9 (10.0–13.0) | 12.0±0.9 (10.0–14.0) | 11.4±1.0 (10.0–13.0) |
| EN | 7.6±0.5 (6.5–8.3) | 7.0±0.6 (6.0–8.3) | 7.6±0.5 (6.8–8.7) | 8.1±0.5 (6.6–8.6) |
| IN | 3.0±0.2 (2.7–3.6) | 3.2±0.3 (2.7–3.7) | 3.2±0.3 (2.7–3.8) | 3.3±0.3 (2.9–3.7) |
| IO | 7.5±0.3 (6.9–8.0) | 6.9±0.4 (6.2–7.3) | 7.4±0.5 (6.4–8.3) | 7.8±0.6 (6.1–8.8) |
| EYE | 5.5±0.3 (4.9–6.1) | 5.0±0.4 (4.4–5.9) | 5.6±0.5 (5.0–6.8) | 5.3±0.4 (4.4–6.1) |
| Trk | 43.9±3.3 (37.6–49.9) | 44.2±3.9 (38.3–52.6) | 46.3±4.3 (38.7–54.8) | 44.7±4.6 (34.1–54.3) |
| ArmL | 11.9±0.6 (10.4–13.0) | 11.1±0.9 (9.6–12.9) | 12.2±0.9 (10.8–13.6) | 12.5±1.0 (8.7–14.1) |
| LegL | 13.7±0.8 (12.2–15.6) | 12.5±0.8 (11.0–14.3) | 14.0±1.0 (11.8–15.9) | 14.3±1.3 (9.9–16.7) |
| 3FW | 2.6±0.2 (2.2–2.8) | 2.3±0.2 (1.7–2.7) | 2.6±0.4 (1.9–3.3) | 2.7±0.3 (1.8–3.3) |
| 3TW | 2.9±0.2 (2.6–3.3) | 2.6±0.3 (2.1–3.0) | 2.9±0.4 (2.1–3.5) | 3.0±0.4 (1.9–3.6) |
| 3FL | 7.7±0.5 (7.0–8.0) | 7.8±0.5 (6.0–8.0) | 8.1±0.5 (7.0–9.0) | 8.4±0.8 (7.0–10.0) |
| 3TL | 8.3±0.5 (8.0–9.0) | 8.1±0.3 (8.0–9.0) | 9.0±0.6 (8.0–10.0) | 9.0±0.5 (8.0–10.0) |
| **Ratios** |  |  |  |  |
| HW/SVL | 0.19±0.01 (0.17–0.20) | 0.18±0.01 (0.17–0.21) | 0.19±0.01 (0.17–0.20) | 0.19±0.01 (0.18–0.21) |
| HD/SVL | 0.08±0.01 (0.07–0.09) | 0.11±0.01 (0.09–0.12) | 0.10±0.01 (0.09–0.11) | 0.10±0.01 (0.09–0.13) |
| HL/SVL | 0.23±0.01 (0.22–0.24) | 0.23±0.01 (0.22–0.25) | 0.23±0.01 (0.22–0.25) | 0.25±0.01 (0.24–0.27) |
| EN/SVL | 0.081±0.006 (0.069–0.090) | 0.081±0.005 (0.072–0.090) | 0.080±0.004 (0.072–0.089) | 0.086±0.003 (0.078–0.093) |
| EYE/SVL | 0.059±0.005 (0.054–0.070) | 0.059±0.006 (0.049–0.068) | 0.059±0.005 (0.049–0.068) | 0.057±0.004 (0.051–0.064) |
| Trk/SVL | 0.47±0.02 (0.43–0.50) | 0.49±0.02 (0.45–0.53) | 0.48±0.02 (0.42–0.51) | 0.47±0.03 (0.42–0.53) |
| ArmL/SVL | 0.13±0.00 (0.12–0.13) | 0.12±0.01 (0.11–0.13) | 0.13±0.01 (0.12–0.14) | 0.13±0.01 (0.12–0.15) |
| LegL/SVL | 0.15±0.01 (0.13–0.16) | 0.14±0.01 (0.12–0.15) | 0.15±0.01 (0.13–0.16) | 0.15±0.01 (0.14–0.17) |
| 3TW/SVL | 0.031±0.002 (0.028–0.034) | 0.030±0.002 (0.023–0.034) | 0.031±0.003 (0.023–0.036) | 0.032±0.003 (0.027–0.036) |
| **Original tail** | n=10 | n =20 | n = 13 | n = 9 |
| TL | 73.2±8.2 (60.0–86.0) | 64.7±5.7 (56.0–77.0) | 65.8±7.2 (57.0–82.0) | 65.8±8.9 (60.0–77.0) |
| TW | 9.0±1.6 (6.3–10.9) | 11.0±1.5 (7.8–13.6) | 10.6±1.4 (8.3–12.9) | 10.1±2.1 (5.8–13.3) |
| TD | 6.1±0.5 (5.3–6.8) | 8.7±1.2 (7.1–11.4) | 7.5±1.4 (5.6–10.1) | 7.7±1.8 (4.9–10.2) |
| TL/SVL | 0.77±0.07 (0.65–0.87) | 0.72±0.05 (0.64–0.79) | 0.70±0.05 (0.61–0.78) | 0.71±0.04 (0.65–0.80) |
| TW/SVL | 0.10±0.02 (0.07–0.11) | 0.12±0.02 (0.10–0.15) | 0.11±0.01 (0.09–0.13) | 0.11±0.01 (0.08–0.14) |
| TW/TL | 0.12±0.02 (0.10–0.15) | 0.17±0.03 (0.14–0.22) | 0.16±0.02 (0.13–0.19) | 0.15±0.01 (0.13–0.17) |
| **Regrown tail** | n=7 | n = 11 | n = 8 | n = 17 |
| TL | 64.0±6.2 (53.0–70.0) | 50.5±6.3 (38.0–58.0) | 68.0±13.0 (27.1–68.0) | 56.8±7.0 (49.0–73.0) |
| TW | 8.7±1.8 (6.1–10.5) | 10.8±1.2 (9.1–12.6) | 11.9±1.0 (8.9–11.9) | 12.3±1.1 (10.4–14.5) |
| TD | 5.9±0.7 (5.0–7.0) | 8.2±0.9 (6.1–9.3) | 10.2±1.0 (7.1–10.2) | 8.8±0.8 (7.5–10.1) |
| TL/SVL | 0.69±0.06 (0.58–0.74) | 0.55±0.05 (0.43–0.62) | 0.72±0.13 (0.30–0.72) | 0.59±0.05 (0.51–0.66) |
| TW/SVL | 0.09±0.02 (0.07–0.11) | 0.12±0.01 (0.10–0.13) | 0.13±0.01 (0.09–0.13) | 0.13±0.01 (0.12–0.15) |
| TW/TL | 0.13±0.02 (0.11–0.16) | 0.22±0.04 (0.16–0.30) | 0.37±0.07 (0.13–0.37) | 0.22±0.02 (0.18–0.26) |

**Appendix S1. Additional material examined.**

***Oedura cincta***

**New South Wales:** AMS R107176, Byrock (30.67°S, 146.40°E); AMS R127130 Barrakee Station, 3.2 km east of Main Entrance, via Bourke-Wanaaring Rd (29.98°S, 145.00°E); AMS R137613, Byerawering Property Homestead (29.13°S, 147.17°E); AMS R137634, Wanaaring, 53 km east of Bourke Rd (29.67°S, 144.60°E); AMS R138446, Bourke-Wanaaring Rd, 1.2 km west Warrego River Bridge (30.00°S, 145.35°E).

**Northern Territory:** AMSR52142, 10 Mi.W.of Stuart Hwy Along Yuendumu Rd (-23.583, 133.566); AMSR52151 Winnecke Goldfields (-23.333, 134.383); AMSR52153, Alice Springs (-23.666, 133.883); AMSR52157–59, Wiggley’s Waterhole, Alice Springs (-23.663, 133.883); AMSR52160, Stuart Hwy, 3, km N. of Alice Springs (-23.600, 133.866); AMS R87680–1, AMS R 87683–4, AMS R87687, Mt Doreen (22.03°S, 131.33°E); AMS R136049, vicinity of 8 Mile Bore, 49.7 km northwest of Yuenduma turnoff on Tanami Road (22.12°S, 131.37°E); CCM5969 Lawrence Gorge (-23.05477, 132.78166); CCM6003–4 Roma Gorge (-23.65595, 132.41298); NTM R1407, Wolfram Hill, 59.6 km west of Yuendumu (22.03°S, 131.33°E); NTM R11413, Jesse Gap, 17 km east of Alice Springs (23.75°S, 134.017°E); NTM R18172, Harts Range, Mt Riddock Station (23.08°S, 134.60°E); NTM R18278, Paddy’s Rockhole (22.40°S, 137.67°E); NTM R35902, NTM R35912, NTM R35919, 10 km south of Mt Doreen ruins (22.11°S, 131.39°E); SAMA R38842–3, Honeymoon Gap, Alice Springs (23.75°S, 133.75°E); SAMA R38844–6, 3 km west of Standley Chasm turnoff on Larapinta Drive (23.77°S, 133.53°E); SAMA R65905–6, Emily Gap (23.74°S, 133.95°E).

**Queensland:** AMS R139806, 9.3 km south of Charleville on Mitchell Hwy (26.49°S, 146.22°E); SAMA R42883–4, 30 km east of Noonbah Station (24.12°S, 143.42°E); SAMA R42894–5, 5 km west of Noonbah Station (24.08°S, 143.13°E); SAMA R42913, 85 km west of Windorah (25.35°S, 141.83°E); SAMA R55903, 9 km north of NSW/QLD border on Mitchell highway (28.96°S, 145.73°E); SAMA R65405, 6.7 km east of Noonbah Homestead (24.10°S, 143.25°E); QM J46669, Victoria Downs, 7 km north of Charleville turnoff, Augathella Road (26.42°S, 147.03°E); QM J71840, QM J71842, Dynevor Downs, 64 km east of Thargomindah, Dynevor Lake (-28.12°S, 144.17); QM J88239, Culgoa Floodplain National Park (28.88°S, 146.99°E); QM J89717, Winton area (22.46°S, 142.97°E); QM J90783, Noonbah Dam (24.10°S, 143.16°E); QM J90798, Wondula House, 0.5 km west of Ballard Tank, Plevna Downs (26.74°S, 142.56°E).

**South Australia:** SAMA R38016, Faraway Bore, Danggali Conservation Park (33.50°S, 140.55°E); SAMA R38900–1, 0.5 km south of Angepena Homestead (30.58°S, 138.85°E); SAMA R50764, 1 km southwest of Mt Serle Homestead (30.53°S, 138.85°E); SAMA R51833, 0.5 km north-northwest of Crump Bore (30.64°S, 138.87°E); SAMA R52203, 2.7 km west of Lance Bore, Narrina Station (30.96°S, 138.77°E); SAMA R52333, 6 km east-southeast of Blackwater Springs, Oratunga Station (31.10°S, 138.88°E); SAMA R54300, west of Bellbird Campsite, Gluepot Reserve (33.70°S, 140.16°E); SAMA R64526, 15.4 km north-northeast of Nantawarrina Homestead (30.70°S, 139.06°E); SAMA R64626, 11.3 km south-southwest of The John Crossing (30.70°S, 139.05°E).

***Oedura filicipoda***

**Western Australia:** WAM R60685, WAM R83707–8 (holotype and paratypes) – Camp Creek, Mitchell Plateau (14.83°S, 125.83); WAM R86897 – 11 km SE Mount Daglish (16.38°S, 124.98*°*E); WAM R167805 – Surveyor’s Pool (14.67°S, 125.73*°*E); WAM R138874 – 4.1 km S Donkin’s Hill (14.99°S, 125.51*°*E); WAM R171552 – Prince Regent Nature Reserve (15.76°S, 125.26*°*E).

***Oedura gemmata***

**Northern Territory:** NMV D72584, NMV D72598–600; NMV D72621, Near Kikiyown (also called ‘Kikikyon’) (12.19°S, 133.81°E); NTM R27383, Oenpelli (12.38°S, 133.02°E); NTM R27599, Kikikyaw (12.19°S, 133.81°E); NTM R34986–7, Mountain Valley Station (13.98°S, 133.18°E); NTM R35680, Kakadu National Park (13.86°S, 132.98°E); NTM R35753, Oenpelli Reservoir (12.38°S, 133.07°E); QM J83987–8, Kolorbidahdah (12.65°S, 134.29°E).

***Oedura marmorata***

**Northern Territory:** NTM R13295, Victoria River Gregory National Park (15.94°S, 130.50°E); NTM R19029–31, North Marchinbar Island (11.28°S, 136.63°E); NTM R21895, Low Hill, Roper River (14.65°S, 134.35°E); NTM R22444, Limmen Gate National Park (15.78°S, 135.33°E); NTM R33814, Bauhinia Downs Station (15.93°S, 135.32°E); NTM R36709, Fish River Station (14.07°S, 130.79°E); NTM R36746, Wongalara (14.13°S, 134.34°E).

***Oedura murrumanu***

**Western Australia:** WAM R173368 (holotype), WAM R173370, NMV D76948 (paratypes), Oscar Range, Western Australia (17.9166°S, 125.3024°E); NMV D77002, NMV D76947, WAM R173369 (paratypes), Oscar Range (17.9125°S, 125.2827°E).
